# Research progress in clinical utilization of H_2_ as a pleiotropic agent

**DOI:** 10.3389/fphar.2026.1884091

**Published:** 2026-09-23

**Authors:** Qinghui Zhao, Tengchong Li, Rongke Lv, Dong Li, Hongru Li

**Affiliations:** 1 Institute of Physical Culture, Huanghuai University, Zhu Madian, China; 2 Zhumadian Central Hospital, Zhu Madian, China

**Keywords:** action mechanisms, anti-inflammatory, antioxidant, clinical utilization, H_2_ medicine, pleiotropic agent

## Abstract

H_2_ is a fundamental environmental factor for the origin of life, providing an energetic basis for the emergence of primitive life. Through its selective antioxidant, anti-inflammatory, and anti-apoptotic activities, as well as its regulatory effects on cell signaling pathways and gene expression, H_2_ exerts broad biological effects and has emerged as a favorable pleiotropic therapeutic agent. To date, hydrogen medicine research has covered almost all types of diseases across organ systems, with more than 170 disease models under investigation. Amid the growing demand for translational applications of hydrogen medicine, clinical research has gradually become a research hotspot. Here, we extensively reviewed the latest mechanistic understanding of H_2_ and comprehensively summarized the progress of clinical studies on H_2_ in diseases of different systems, with the goal of informing future large-scale, multi-center clinical trials and supporting the multidisciplinary development of H_2_ medicine.

## 1 Introduction

H_2_ is a fundamental environmental factor in the origin of life. Since the 1940s, pioneers in the European diving medical community have taken the lead in conducting a series of studies on the application of H_2_ in deep-sea diving (Harris et al., 2024). Subsequent research has found that even under the condition of high-pressure H_2_ at dozens of atmospheres, there is almost no toxicity except for an inhibitory effect on heart rate and a slight anesthetic effect on the central nervous system (Tong et al., 2025). As early as 1975, American scholars published an article in Science, demonstrating that hydrogen inhalation (HI) at 8 atm (1 atm = 1.013 × 10^5^ Pa) could treat squamous cell carcinoma of the skin in mice (Dole et al., 1975). However, this study did not attract the attention of the academic community. The main reason might be that considering the risk of deflagration associated with the use of high-pressure H_2_, clinical application was almost unfeasible. It was not until 2007 (Ohsawa et al., 2007) published a report in Nature Medicine, stating that inhaling 2% H_2_ could trigger biological effects and pointing out that H_2_ acts as a selective antioxidant. Its medical value was quickly recognized. In the past 19 years, the biomedical effects of H_2_ have been extensively studied. More than 2,000 peer-reviewed papers have been published successively, and a unique theoretical system of H_2_ medicine has been established. Preclinical studies have shown that H_2_ has significant therapeutic effects in various disease models, including ovarian cancer, type 2 diabetes, ischemia-reperfusion injury, and brain injury in rodents (Todorović and Ostojic, 2025; Zhang et al., 2025a). In addition to preclinical studies, a large number of clinical studies have also evaluated the potential effects of H_2_ in different situations, such as oncology, metabolic disorders (e.g., hyperuricemia, type 2 diabetes, obesity, metabolic syndrome), neurological diseases, skin-related diseases, and diseases associated with oxidative stress and inflammation (Johnsen et al., 2023). This review aims to summarize the current evidence regarding the clinical application of H_2_, providing a basis for future large-scale, multi-center trials and mechanistic research to further clarify its therapeutic potential. To objectively evaluate the reliability of existing research on hydrogen therapy against tumors, all retrieved literatures were graded according to study design and evidence quality. We referenced and adopted the seven-tier hierarchy of scientific evidence for evidence-based clinical practice published by Duke University Medical Center Library in 2022, with evidence levels ranked from highest to lowest. Level 1 corresponds to meta-analyses of randomized controlled clinical trials, representing the highest level of clinical evidence. If the analysis yields statistically significant results, the relevant intervention can be confirmed to be clinically effective. Level 2 refers to systematic reviews that synthesize multiple randomized controlled clinical trials, which are high-quality synthesized evidence. Level 3 includes individual prospective randomized controlled clinical trials, regarded as the gold standard for single clinical studies. Levels 4 to 6 cover a variety of observational studies without random allocation or placebo control, which cannot verify causal relationships between variables, including cohort studies, case-control studies, cross-sectional descriptive studies, and so on. Level 7 is the lowest tier of evidence, consisting of non-human studies such as animal experiments and *in vitro* basic experiments, which can only be used as preliminary references for clinical research (Duke University Medical Center Library and Archives, 2022; Gibb et al., 2023) (Figure 1).

**FIGURE 1:**
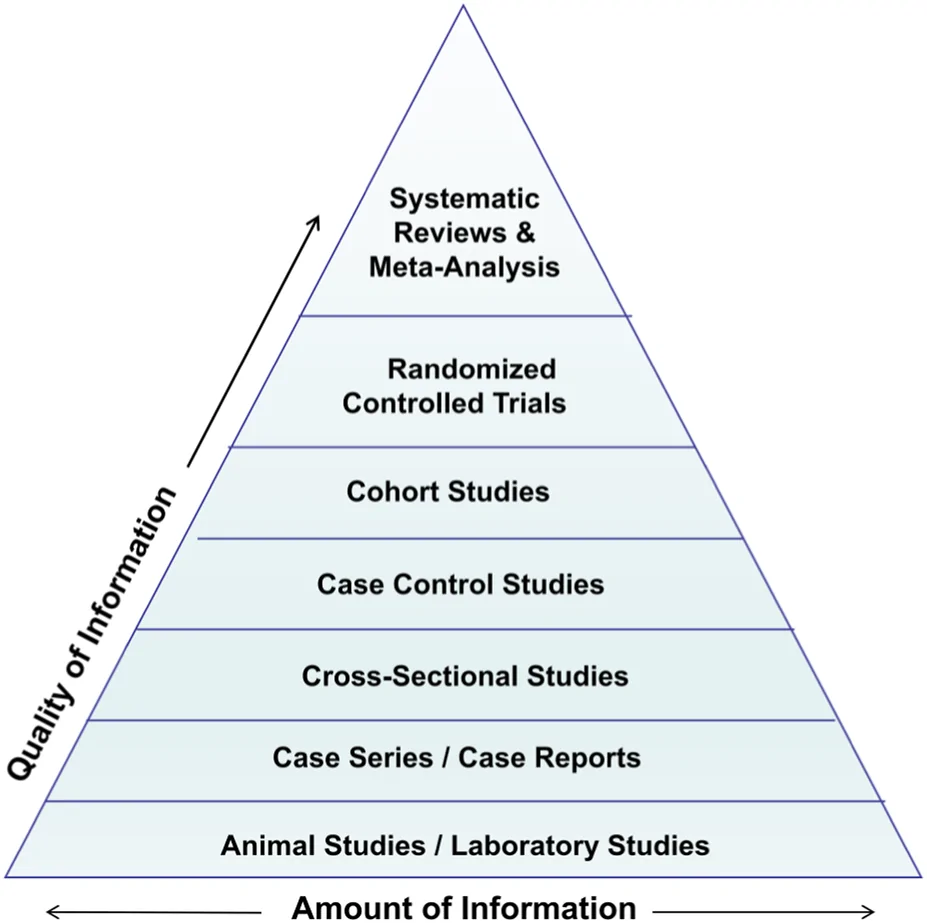
Evaluation of available clinical evidence.

## 2 Physicochemical properties of H_2_

H_2_ is located at the top of the periodic table of elements and is an important substance involved in the evolution of the universe and the origin of life. Since the birth of the universe, H_2_ has always been present throughout the entire process of cosmic evolution and occupied an extremely high proportion in the original Earth’s atmosphere, being a key environmental condition for the origin of life (Young et al., 2023). A series of pre-life chemical evolution studies, represented by the Miller experiment, have shown that hydrogen is an indispensable material basis in the evolution process of organic molecules on the original Earth. As the simplest reducing substrate, H_2_ can provide important energy support for the formation and emergence of primitive life. In 2007, H_2_ were confirmed to have selective antioxidant biological characteristics, and since then, they have become a research hotspot in the field of biomedicine. Due to their physical and chemical characteristics such as small particle size, non-polarity, and high diffusivity, H_2_ can freely penetrate the cell membrane and enter the mitochondria and nucleus, widely participating in various physiological regulatory processes of the body (Ohta, 2015). In terms of safety characteristics, H_2_ has a wide range of flammability and explosiveness limits. Its volume explosion limit in air is 4%–75% (Carcassi and Fineschi, 2005), and H_2_ mixed gas within this concentration range will ignite and explode upon contact with fire. Therefore, to ensure the safety of experiments and applications, currently, a hydrogen concentration of 2%–4% is commonly used for inhalation intervention in the scientific research field. H_2_ has a certain solubility in water. Under standard conditions (20 °C, 101.325 kPa), 100 mL of water can dissolve 1.83 mL of H_2_, corresponding to a dissolution concentration of approximately 0.8 mmol/L (1.6 mg/L, 1.6 ppm). Oral consumption of hydrogen-rich water (HRW) is an important way to supplement H_2_ in the body. Current experimental studies mostly use HRW a concentration of no less than 0.6 mmol/L for related experiments (Han et al., 2015). Currently, the common preparation methods of HRW include: directly introducing H_2_ into the water body (with the addition of pressurization technology to enhance the hydrogen solubility effect) (Nagata et al., 2009), electrolyzing water to dissolve hydrogen in water, and preparing HRW through the chemical reaction between metal magnesium (Mg) and water (Song et al., 2013). Among them, the micro-nano bubble-assisted hydrogen solubilization technology can increase the hydrogen concentration in water to above 2 mmol/L, which is an efficient physical solubilization method (Kikuchi et al., 2006). However, due to the extremely small particle size and strong penetration of H_2_, their physical characteristics also lead to the easy evaporation of dissolved H_2_ from water, making the storage and preservation of HRW a key technical problem. In an open environment, the hydrogen concentration in saturated HRW will decay by 50% after 2 h and almost completely dissipate from the water after 10 h (Hou et al., 2016). Based on this characteristic, currently, most commercial HRW products use aluminum packaging for sealed storage to effectively inhibit hydrogen evaporation and maintain the stability of the solution’s H_2_ concentration.

## 3 H_2_ administration methods

### 3.1 Inhalation and intraperitoneal gaseous H_2_ administration

The H_2_ administration methods used in the relevant studies include HI, drinking HRW, drop infusion of H_2_-rich saline (HRS), HRW bathing, etc. The choice of H_2_ administration method mainly depends on the specific research purpose.

The earliest HI was applied in the field of diving medicine, mainly to increase the diving depth and avoid nitrogen anesthesia by choosing hydrogen-oxygen mixed gas as the breathing medium. Although this was not for treating diseases, it was the first to confirm the safety of HI. Currently, hydrogen electrolysis water generators can be used to simultaneously provide oxygen and H_2_ to patients through masks or nasal tubes. This method has a fast reaction and direct administration, but the effect of continuous inhalation is not ideal. Studies on various disease models have shown that 2%–4% low-concentration hydrogen-containing gas can reach an effective therapeutic concentration (Yuan et al., 2023; Ono et al., 2017). Intraperitoneal injection: in animal models, the direct intraperitoneal injection of H_2_ is used. Compared with direct inhalation, this method is simpler and can maintain a high H_2_ concentration for a longer time (Chen Y. et al., 2025).

### 3.2 Administration via hydrogen-rich liquid preparations

HRW is convenient to prepare and has high safety, making it more suitable for clinical application and promotion. Currently, many clinical studies have confirmed the protective effect of HRW. In addition, compared with other hydrogen administration methods, drinking HRW can markedly increase the local hydrogen concentration in the gastrointestinal system (Yuan et al., 2023), which may be more suitable for studies related to gastrointestinal diseases. The hydrogen-rich 0.9% sodium chloride solution for injection is easier to control the H_2_ concentration than HRW for oral administration, and it is also cheaper, more convenient, and safer, and improves hydrogen utilization. Administration routes include intraperitoneal, intravenous, intrathecal, and intravitreal injections, with intraperitoneal injection being the most common.

H_2_ can also be dissolved in physiological saline. Studies have shown that using hydrogen-rich physiological saline for cryopreservation of transplanted organs can reduce organ damage caused by ischemia and reperfusion and reduce the risk of organ transplantation (Saito et al., 2020; Quan et al., 2021); hydrogen-rich physiological saline can also be used as an irrigation solution for cataract surgery to protect corneal endothelial cells (Li et al., 2023; Igarashi et al., 2019). In addition, because hydrogen easily penetrates the skin and distributes throughout the body through blood flow, HRW showering can also be used. However, due to the low solubility of H_2_, it is easily dispersed in the air, with low H_2_ absorption efficiency, often requiring large doses but with low absorption, and it is difficult to conduct quantitative research.

### 3.3 Novel sustained hydrogen-releasing biomaterial-based delivery

Direct H_2_ intake is simple and quick, but its effective duration is short, and it is difficult for H_2_ to reach the lesion site. The hydrogen release system constructed based on micro-nanomaterials can continuously release H_2_, which is conducive to long-term and precise drug administration. Wan et al. (2018) prepared Mg@PLGA microparticles and injected them into the knee joint of mice with osteoarthritis. Through the Mg-mediated activation/deactivation cycle in body fluids, H_2_ was continuously released *in situ* to alleviate tissue inflammation and prevent cartilage degeneration. Wang et al. (2023) prepared MgH_2_-PLGA microneedle patches that react with body fluids, generating H_2_ that reduced Reactive oxygen species (ROS) production and changes the pathological microenvironment of diabetic patients, helping to accelerate wound healing. Nan et al. (2014) modified the surface structure of metallic Mg through alkaline-thermal treatment to control the degradation rate and H_2_ release rate of Mg, and the continuous release of H_2_ was conducive to the long-term elimination of free radicals and the inhibition of tumor growth. Compared with normal cells, tumor cells have a high aerobic glycolysis rate, produce large amounts of lactic acid, and have a low extracellular fluid pH. These characteristics are conducive to the targeted treatment of tumors. Yang et al. (2022) prepared ammonia borane nanoparticles (AB@MSN) which not only have a high hydrogen-loading capacity but also exhibit a high acid response. In normal body fluids, they do not produce H_2_. These nanoparticles can effectively inhibit tumor growth without affecting normal cells and tissues, and have high biological safety. Zhang et al. (2023) prepared a Mg-containing microsphere (Mg@PLPEMS) that responds to ROS. This microsphere can control H_2_ release and remove hydrogen peroxide (H_2_O_2_), and can also reduce the degradation of extracellular matrix and the apoptosis of intervertebral disc cells. It has a marked antioxidant and anti-inflammatory effect on intervertebral disc degeneration rats. In addition, light-triggered drug delivery is easy to operate and can be administered on demand. For example, Zhao et al. (2021) developed a nano-catalyst that generates H_2_ through near-infrared light catalysis. The holes generated can consume the overexpressed glutathione in tumor cells, and the generated H_2_ and consumed glutathione reduce the energy of cancer cells and disrupt the redox balance, thereby inducing tumor cell apoptosis. The solubility of H_2_ in the brain of Alzheimer’s Disease (AD) patients is low, and it is difficult to accumulate continuously and efficiently. Zhang et al. (2019) developed PdH nanoparticles with high hydrogen-loading capacity and can continuously release H_2_ in the AD brain. Due to the catalytic hydrogenation effect of Pd, the H_2_ released by PdH nanoparticles has high biological reducibility, which is conducive to the efficient removal of free radicals in a self-catalytic manner, reduces the production and aggregation of amyloid proteins, restores mitochondrial function, and blocks synaptic and neuronal apoptosis.

### 3.4 Endogenous H_2_ production induction

In addition to the aforementioned exogenous H_2_ supply pathways, stimulating the production of endogenous hydrogen can also achieve the purpose of H_2_ supply. Endogenous H_2_ in the human body is derived from bacterial fermentation in the body, mainly from the intestines. It is convenient to administer and has a fast H_2_ production rate. Specific methods include oral administration of drugs and dietary promotion (Yuan et al., 2023; Chen et al., 2022). However, due to the notable individual differences in the intestinal microbiota, it is difficult to control the dosage.

### 3.5 Heterogeneity and standardization challenges of H_2_ administration

Substantial inconsistencies can be found in existing H_2_ administration schemes, such as inconsistent inhalation concentrations, disparate treatment durations, diverse preparation procedures of HRW and unstable dissolved H_2_ content, alongside variable administration routes and dosing intervals. Such discrepancies make it difficult to compare outcomes across independent studies. From a pharmacokinetic perspective, each delivery route displays distinct *in vivo* behaviors. Inhaled H_2_ distributes rapidly throughout the body yet acts transiently; oral HRW mainly accumulates within the gastrointestinal tract, whereas injection sustains high local H_2_ concentrations in target tissues. To date, no solid evidence supports bioequivalence between oral intake and gas inhalation, and their tissue penetration profiles remain poorly defined. Variations in preparation and storage further destabilize dissolved H_2_ levels in liquid formulations, resulting in ambiguous dose-effect correlations in available preclinical and clinical trials. Besides, disease-specific effective and safe therapeutic ranges have not yet been determined. Overall, systematic research resolving these key challenges is still lacking, and further pharmacokinetic exploration is essential to standardize H_2_ application and objectively evaluate the merits of distinct administration approaches (Figure 2).

**FIGURE 2:**
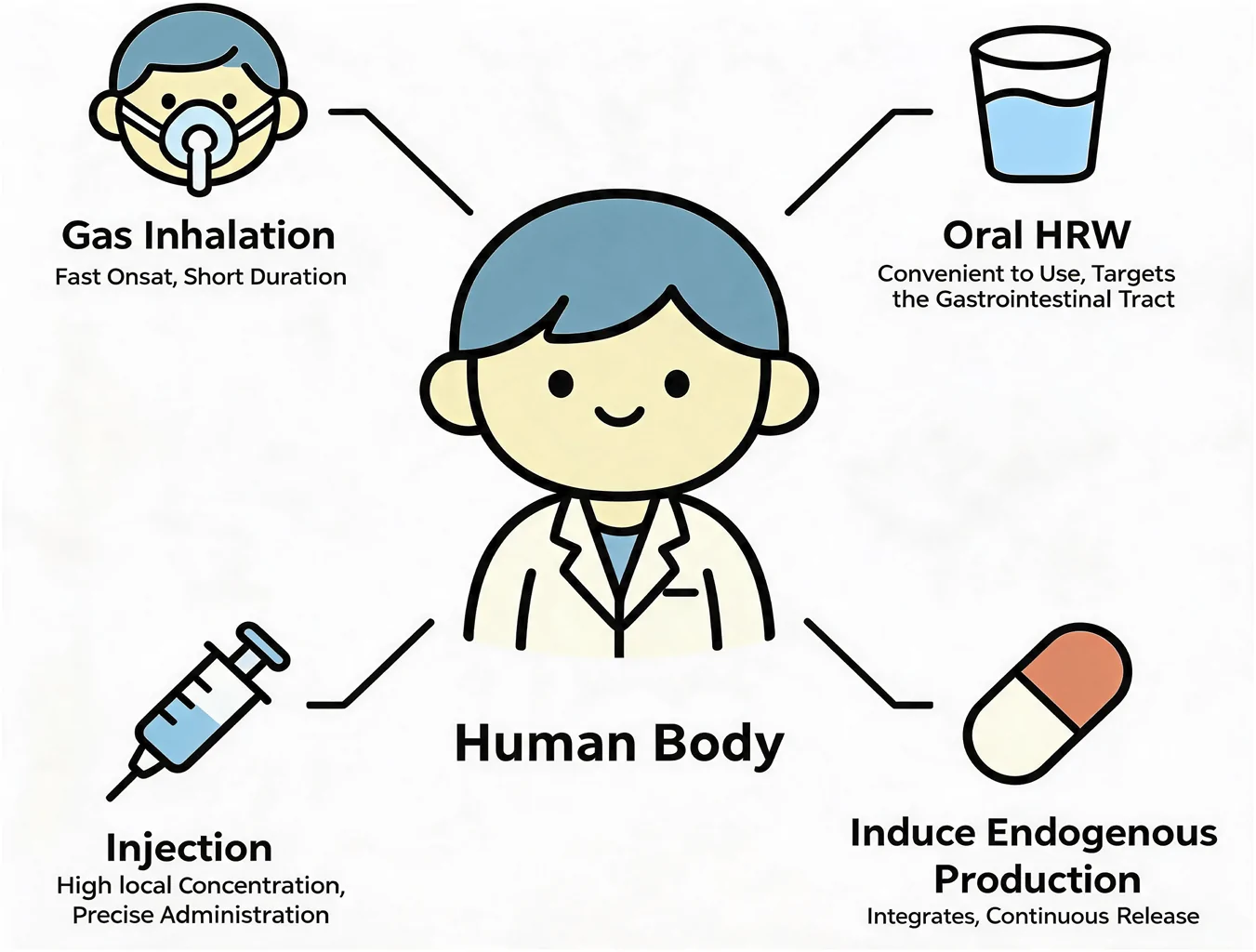
Comparison of hydrogen delivery routes.

## 4 Main mechanisms of action of H_2_

### 4.1 Antioxidant effect

ROS are a group of reactive molecules derived from superoxide anion (O_2_^−^) or H_2_O_2_ (Averill-Bates, 2024). Multiple subcellular compartments, including mitochondria, the endoplasmic reticulum, lysosomes, and the plasma membrane, can generate and release ROS (Averill-Bates 2024; Degechisa et al., 2022). Endogenous ROS arise mainly from oxidative phosphorylation in the mitochondrial respiratory chain, from catalytic reactions mediated by cytoplasmic NADPH oxidase, and from systems such as xanthine oxidase and uncoupled nitric oxide synthase (Averill-Bates, 2024). Exogenous ROS can be induced by drugs, chemical pollutants, and radiotherapy (Zhang et al., 2022; Cao Z. et al., 2025). At physiological levels, ROS are essential for diverse cellular processes, a state often termed “oxidative eustress” (Averill-Bates, 2024). For instance, H_2_O_2_ activates several signaling pathways, including mitogen-activated protein kinase (MAPK), phosphatidylinositol 3-kinase (PI3K), and protein kinase B (Akt) (Guo and Dong, 2022; Płóciniczak et al., 2025). ROS also activate transcription factors such as activator protein 1, nuclear factor-κB (NF-κB), nuclear factor erythroid 2-related factor 2 (Nrf2), and hypoxia-inducible factor 1α (HIF-1α). Moreover, physiological concentrations of ROS stimulate vascular endothelial growth factor (VEGF) and thereby promote angiogenesis. However, excessive ROS accumulation causes oxidative damage to DNA, proteins, and lipids, disrupts redox homeostasis, and serves as a risk factor for many diseases (Averill-Bates 2024; Palma et al., 2024).

The antioxidant actions of H_2_ can be summarized as follows. First, H_2_ reacts non-enzymatically with free radicals via random collisions. This interaction is highly selective: H_2_ readily reacts with strong oxidants such as hydroxyl radical (·OH) and peroxynitrite (ONOO^−^), thereby preventing them from damaging nucleic acids, lipids, and proteins. In contrast, H_2_ shows low reactivity toward weaker signaling-related oxidants like superoxide, nitric oxide, and H_2_O_2_, leaving normal cell signaling largely unaffected. This selective antioxidant property gives H_2_ certain advantages over common antioxidants such as vitamins C and E (Ria et al., 2025; Russell, 2025). Second, H_2_ can lower ROS levels directly or as a modulator of gas-mediated signals; it does so by upregulating superoxide dismutase (SOD) and glutathione (GSH), and by downregulating NADPH oxidase (NOX2) expression (Jin et al., 2025; Zhao et al., 2019). Another study (Ohno et al., 2012) showed that H_2_ primarily inhibits ASK1 phosphorylation and the downstream signaling molecules p38 MAPK or c-Jun N-terminal kinase (JNK), without affecting ROS produced by NADPH oxidase (Figure 3).

**FIGURE 3:**
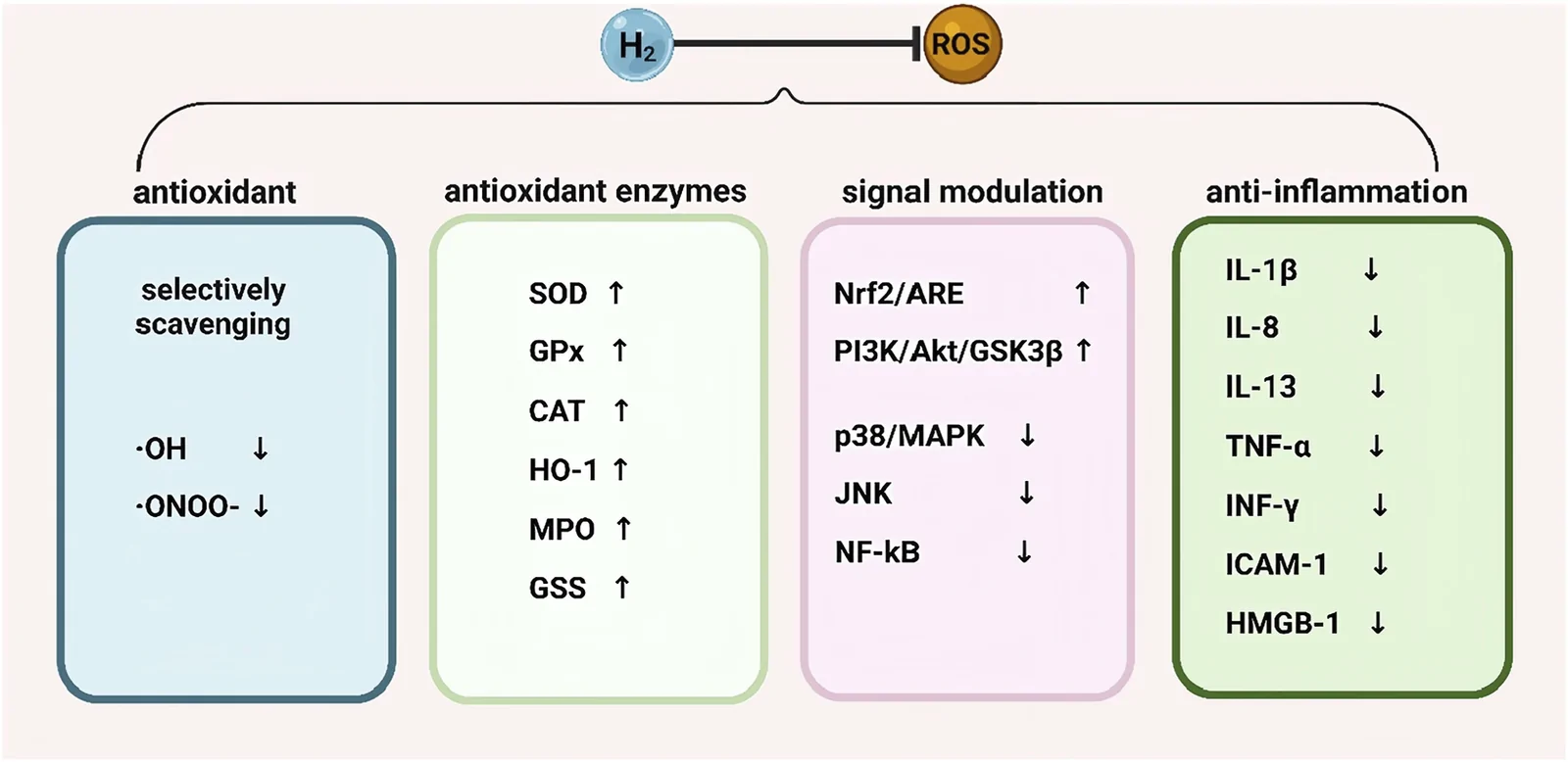
Mechanisms of H_2_ in anti-oxidative stress (reprinted with permission from Zhou et al., 2024, licensed under CC BY 4.0).

The impact on the lipid peroxidation free radical chain reaction may represent another crucial mechanism underlying the antioxidant effect of H_2_ (Ichihara et al., 2015). Multiple studies have confirmed that H_2_ can safeguard cells against lipid and fatty acid peroxidation damage (Iuchi et al., 2019; Yang et al., 2020). 4-hydroxy-2-nonenal (4-HNE), a reactive aldehyde, is a by-product generated from ROS-driven lipid peroxidation and is associated with tissue injury, dysfunction, age-related damage, and other pathological conditions including cancer, AD, diabetes, cardiovascular and inflammatory complications. Considered a marker of oxidative stress, HNE also functions as a secondary signaling molecule that regulates multiple cellular signaling pathways; its biological activity relies on intracellular concentration, which differentially modulates cell death, proliferation and differentiation. Accordingly, mechanisms maintaining intracellular HNE levels are not only critical for resistance to oxidative stress but also participate importantly in the pathogenesis of various diseases (Shoeb et al., 2014; Ioannidis et al., 2025). Relevant studies have demonstrated that H_2_ exerts inhibitory effects on oxidative damage by lowering the levels of inducible nitric oxide synthase (i-NOS) and 4-HNE, implying that H_2_ may help prevent or mitigate risks of oxidative stress-related diseases (Song et al., 2023; Roch et al., 2022; Fujita et al., 2009; Martínez-Marte et al., 2024; Terasaki et al., 2019).

Moreover, H_2_ can also reduce the expression of myeloperoxidase (MPO), inhibit the activity of mitochondrial oxidoreductase, and stabilize the mitochondrial membrane potential, thereby alleviating tissue damage caused by oxidative stress (Yang et al., 2020; Zhou et al., 2024) (Figure 2). In addition, H_2_ therapy was associated with substantial reductions in NF-κB, heat shock protein 70, and matrix metalloproteinase-9 (MMP-9) levels (Kura et al., 2022). HIF-1α participates in the regulation of pathological processes through multiple signaling pathways, including PI3K/Akt/mTOR, MAPK/ERK, NF-κB, JAK/STAT3, and Wnt/β-catenin. These pathways are intertwined to form a homeostatic regulatory network, enabling cells to dynamically respond to changes in oxygen concentration. In acute hypoxia (e.g., cerebral infarction and acute myocarditis), HIF-1α is rapidly upregulated, activating genes related to angiogenesis and energy metabolism (such as VEGF, EPO, and LDHA) to promote tissue adaptation and repair. In chronic hypoxia (e.g., vascular dementia, COPD, chronic heart failure, and diabetic nephropathy), HIF-1α maintains a moderate upregulation, reflecting the body’s compensatory metabolic reprogramming under long-term stress. HIF-1α exhibits a “dual character” in hypoxia-related diseases: on one hand, its activation promotes cell survival and angiogenesis, an important part of the body’s self-protection; on the other hand, long-term high expression may lead to pathological angiogenesis, inflammatory responses, and metabolic disorders. Therefore, HIF-1α is both a marker of disease progression and a potential therapeutic target. Guo et al. (2024) found that H_2_ protects the retinas of oxygen-induced retinopathy (OIR) mice from retinopathy by bidirectionally regulating the HIF-1α-VEGF pathway during the hyperoxia and hypoxia phases. Under normal conditions, Nrf2 is bound to its inhibitor Keap1 and remains inactive in the cytoplasm, where it is rapidly degraded via the ubiquitin-proteasome pathway, ensuring low basal transcriptional activity (Guo et al., 2024). Upon stimulation by ROS or other electrophiles, Nrf2 dissociates from Keap1, translocates to the nucleus, forms a heterodimer with Maf proteins, binds to the antioxidant response element (ARE), and activates the transcription of target genes, including phase II metabolic enzymes, antioxidant enzymes, and drug transporters, thereby counteracting oxidative damage. Nrf2 is considered a key transcription factor that regulates cellular defense against xenobiotics and oxidative stress; however, impaired activation or deficiency of Nrf2 increases cellular susceptibility to stress, and Nrf2 has also been implicated in chemical carcinogenesis, prolonged inflammatory repair, and apoptosis. In the developing retina of the OIR model, H_2_ not only inhibits pathological neovascularization but also promotes revascularization, accompanied by increased expression of Nrf2 and its target genes NQO1 and HO-1. Activation of the Nrf2/HO-1 pathway alleviates ischemia-reperfusion injury and improves neurological function. H_2_ activates Nrf2 and its downstream target enzymes, enhancing the activity of endogenous antioxidant enzymes and thereby exerting antioxidant effects (Russell, 2025; Li et al., 2019; Lu et al., 2020; Guo et al., 2024).

### 4.2 Inhibition of inflammatory response

Macrophages possess high plasticity and heterogeneity and are mainly classified into two polarized phenotypes: classically activated M1-type and alternatively activated M2-type macrophages (Chen S. et al., 2025). Generally, M1-type macrophages exert anti-tumor effects by secreting pro-inflammatory factors including TNF-α and CXCL-9 to trigger immune responses, inhibit the proliferation and metastasis of tumor cells, and directly kill neoplastic cells, whereas M2-type macrophages exhibit pro-tumor properties that promote tumor cell proliferation, invasion and metastasis, participate in tumor angiogenesis, suppress immune reactions, and thereby establish a favorable microenvironment for tumor growth (Wang et al., 2023). H_2_ eliminates ROS in immune cells to protect these cells from oxidative stress and further facilitates dendritic cell maturation, T-cell activation and macrophage polarization, consequently mediating anti-tumor activities; additionally, H_2_ attenuates tissue injury by directly downregulating pro-inflammatory cytokines and preventing the infiltration of neutrophils and macrophages (Yin et al., 2022; Xu et al., 2024; Aoki et al., 2022; Zuo et al., 2024; Chen et al., 2024), and its anti-inflammatory effects may also be associated with the regulation of cellular signal transduction and gene expression. Integrated transcriptomic microarray analyses of multiple organs (lung, liver and intestine) from septic mice have demonstrated that H_2_ markedly modulates various signaling pathways during the acute inflammatory phase (Matsuura et al., 2021). With respect to immunomodulation, H_2_ alleviates inflammatory responses in autoimmune diseases such as rheumatoid arthritis, dermatomyositis and psoriasis, improves the disrupted T helper 1 (Th1)/T helper 2 (Th2) balance, and increases the number of CD4^+^CD25^+^Foxp3^+^ regulatory T cells (Tregs), which in turn mitigates tissue damage in allergic rhinitis and cerebral ischemia-reperfusion models; these effects are potentially related to the upregulated expression of TGF-β1 and the downregulated expression of miR-21 and miR-210 (Yang et al., 2020). Moreover, H_2_ activates peroxisome proliferator-activated receptor γ coactivator-1α (PGC-1α), yet the exact mechanisms by which H_2_ acts on specific T-cell subsets remain to be clarified (Yang et al., 2020).

### 4.3 Various effects of H_2_ on cell death

H_2_ exerts bidirectional regulatory effects on cell apoptosis, which predominantly inhibits apoptosis in normal cells. As a typical form of programmed cell death, apoptosis can be modulated by H_2_ via altering the expression levels of apoptosis-related proteins. Specifically, H_2_ downregulates pro-apoptotic factors including Bax, caspase-3, caspase-8 and caspase-12, while upregulating anti-apoptotic Bcl-2 and Bcl-xl (Ma et al., 2025; Meng et al., 2025; Li et al., 2022; Li and Ai, 2017). Terasaki et al. verified that H_2_ inhibits mitochondrial translocation and transcriptional expression of Bax to block apoptotic cascade responses (Terasaki et al., 2011). Mechanistically, H_2_ activates the PI3K/AKT and JAK2-STAT3 signaling pathways in myocardial ischemia-reperfusion injured rats, and suppresses p38 MAPK pathway activation to alleviate LPS-induced acute lung injury and cerebral ischemia-reperfusion injury (Yang et al., 2020; Pan et al., 2025). Notably, H_2_ presents cell-type-dependent apoptotic regulation. Existing studies have proven that H_2_ suppresses proliferation, migration and invasion, and facilitates apoptosis in A549 and H1975 lung cancer cells (Khiji et al., 2024; Yang et al., 2020). Similarly, H_2_ induces apoptotic death in KYSE-70 esophageal squamous cell carcinoma cells, indicating that H_2_ protects normal somatic cells and exerts anti-tumor effects concurrently (Yang et al., 2020; Chu et al., 2025).

Necroptosis, a form of programmed necrotic cell death, serves as a gatekeeper of host defense against certain pathogen invasions, and its dysregulation is also a key factor in many inflammatory diseases (Yan et al., 2022). In a rat model of glycerol-induced acute kidney injury (AKI), the mRNA expression levels of key necroptotic molecules, RIPK3 and MLKL, were considerably elevated in renal tissue. Notably, inhalation of low (4%) or high (67%) concentrations of H_2_ markedly reduced the expression of both RIPK3 and MLKL. The discussion in the original study indicated that the TNF-α/RIPK3/MLKL axis is the primary mediator of necroptosis in tubular injury during glycerol-induced AKI, and that H_2_ inhalation reverses this process by downregulating RIPK3 and MLKL expression. Therefore, H_2_ exerts an inhibitory effect on necroptosis of renal tubular epithelial cells in the pathogenesis of AKI (Xue et al., 2023).

H_2_ mediates dual modulation of autophagy under different pathological conditions. Moderate autophagy maintains intracellular energy homeostasis, whereas excessive or stress-triggered autophagy exacerbates inflammatory injury in multiple organs and tissues (Meng et al., 2025). In sepsis models, H_2_ restrains p-mTOR/mTOR and p62 expression, and elevates the levels of pAMPK/AMPK, LC3II/LC3I, TREM-2 and Beclin-1 to activate protective autophagy, thereby ameliorating neuroinflammation in glial cells (Yang et al., 2020). H_2_ also mitigates chronic intermittent hypoxia-induced renal damage via inhibiting oxidative stress-dependent p38 and JNK MAPKs to initiate autophagy (Yang et al., 2020). By contrast, H_2_ inhibits excessive autophagy to protect pulmonary tissue against LPS-mediated lung damage. It elevates Nrf2-mediated HO-1 expression, activates mTOR signaling, and decreases Beclin-1 and LC3 expression to eliminate excess intracellular reactive oxygen free radicals (Yang et al., 2020). TLR4 is regarded as a potential target for H_2_-modulated autophagy. LPS-activated TLR4 triggers macrophage autophagy, and the inhibitory effect of H_2_ on LPS-induced inflammation further confirms that TLR signaling participates in hydrogen-regulated autophagic processes (Yang et al., 2020).

Pyroptosis is an inflammation-dominated programmed cell death pathway. Caspase-1/4/5 in humans and caspase-1/11 in mice are core initiators of pyroptosis, which defends hosts against pathogenic infection under physiological conditions. Nevertheless, uncontrolled pyroptosis contributes to sepsis and septic shock (Liu et al., 2024). H_2_ has been validated to relieve pyroptosis-associated inflammatory responses. Inhalation of 2% H_2_ decreases caspase-1 expression, and reduces the release of downstream pro-inflammatory IL-1β and IL-18 (Chen Y. et al., 2025). H_2_ upregulates Atg7, and inhibits HMGB1 and IFN-γ expression to block caspase-11-dependent pyroptosis (Yang et al., 2020). Caspase-3 is modulated by H_2_ and participates in both apoptosis and pyroptosis via cleaving gasdermin D, while the interactive mechanism between the two death pathways remains poorly understood (Yang et al., 2020). IFI16-mediated pyroptosis mediates CD4^+^ T cell exhaustion during HIV infection, hence H_2_-targeted pyroptosis regulation is considered a favorable adjuvant strategy for HIV treatment (Yang et al., 2020).

Ferroptosis is an iron-dependent regulated cell death characterized by excessive lipid hydroperoxide accumulation (Mishima et al., 2025; Tong et al., 2026). H_2_ primarily confers anti-ferroptotic effects via restoring intracellular redox balance, inhibiting MAPK signaling, downregulating ferroptosis promoter HMGB1, and enhancing HO-1 activity (Yang et al., 2020; Dai et al., 2026). HO-1 deficiency elevates the susceptibility of renal epithelial cells to ferroptosis, confirming the protective role of HO-1 against ferroptotic damage. Nonetheless, H_2_ exhibits context-dependent effects on ferroptosis. Hydrogen-downregulated miR-9 alleviates ferroptotic injury, whereas the detailed regulatory network remains unclarified (Yang et al., 2020). H_2_ shares similar anti-inflammatory and anti-lipid peroxidation properties with GPX4, and combined application of H_2_ and GPX4 activators provides novel therapeutic insight for lipid peroxidation-related disorders (Tong et al., 2026). Additionally, gut microbiota-derived endogenous H_2_ production exhibits circadian rhythmic oscillation, accompanied by rhythmic alterations of systemic oxidative stress levels (Yang et al., 2020). Given the validated antioxidant capacity of H_2_, H_2_ is speculated to rhythmically modulate ROS homeostasis via circadian clock, though sufficient experimental evidence is still lacking to verify this regulatory mechanism (Yang et al., 2020) (Figure 4).

**FIGURE 4:**
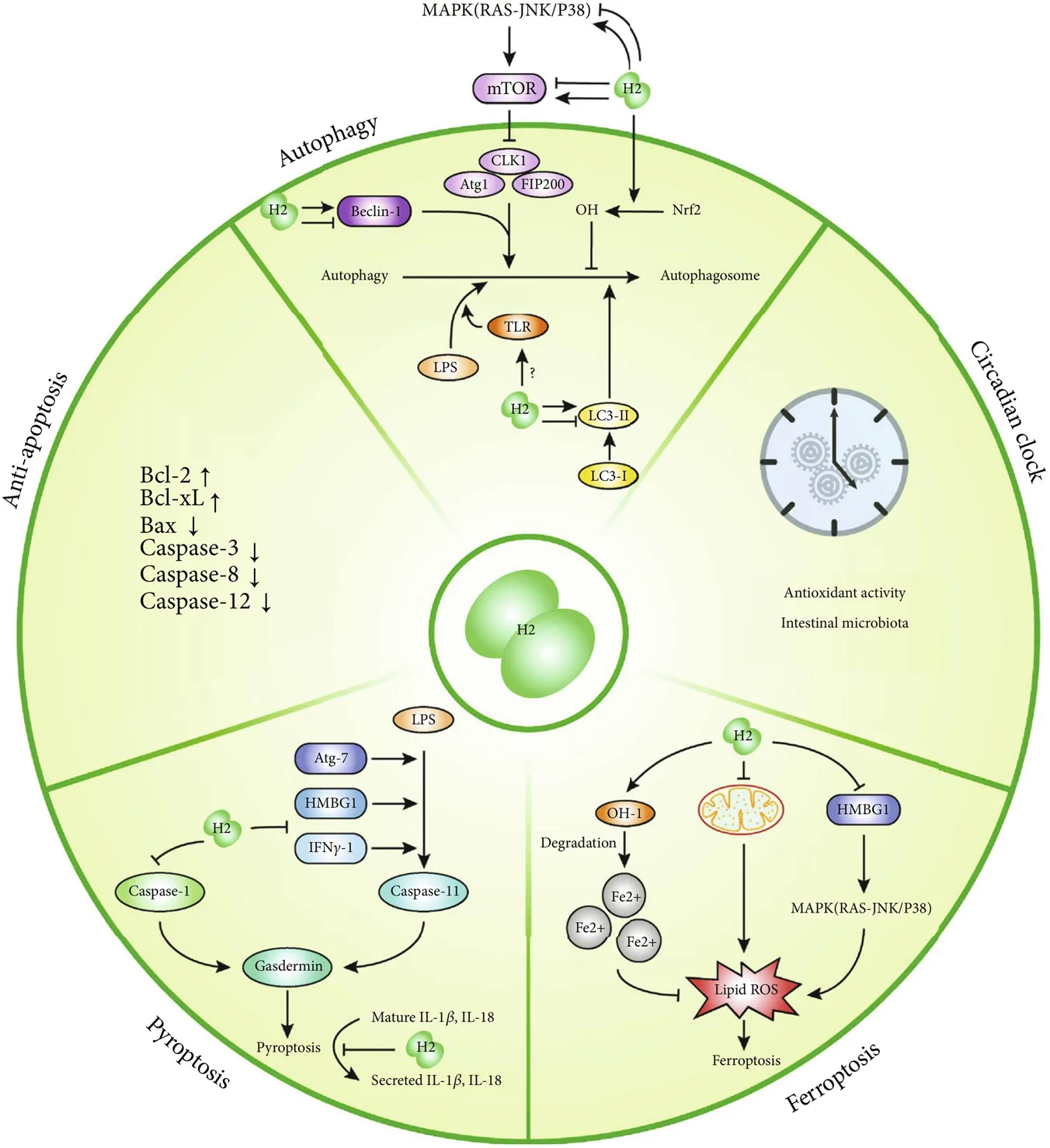
Activities of H_2_ include inhibition of apoptosis, and the regulation of autophagy, circadian clock, ferroptosis, and pyroptosis (reprinted with permission from Yang et al., 2020, licensed under CC BY 4.0).

### 4.4 Regulation of cell signaling pathways and energy metabolism

H_2_ can exert biological effects by influencing cell signaling pathways and gene expression. Some studies have found that H_2_ can increase the expression of mitochondrial genes and the activity of the electron transport chain in cells by inducing the expression of factors related to the mitochondrial unfolded protein response (mtUPR) (ATF5, p-eIF2α, HSP60), thus promoting cell proliferation and differentiation (Hasegawa et al., 2022). In addition, H_2_ can regulate human immune cells such as Th17, Th1, and CD8^+^ cells, reduce the production of cytokines (IL-26 and IFN-γ), improve immune tolerance during pregnancy in pregnant women, and reduce the preterm birth rate (Aoki et al., 2022). By inhibiting HIF-1α-induced glycolysis and alleviating mitochondrial oxidative stress, H_2_ can reduce the proportion of Ptgs2+Nos2+ pro-inflammatory macrophages (PIMs), reduce the harmful effects of PIMs on dermal fibroblasts, and promote extracellular matrix production and wound repair (Xu et al., 2025). Moreover, H_2_ may promote the expression of energy-metabolism-related regulatory factors such as fibroblast growth factor 21 (FGF21), peroxisome proliferator-activated receptor γ co-activator 1α (PGC-1α), uncoupling protein 1 (UCP1), and obesity-related hormones/neuropeptides, thereby promoting the consumption of fatty acids and glucose and stimulating energy metabolism (Kamimura et al., 2016; Tamura et al., 2016).

### 4.5 Targets of H_2_ action: mechanisms and hypotheses

As the evidence of the effectiveness of H_2_ continues to accumulate, the question arises as to what the specific molecular targets of H_2_ action are. It is widely considered that H_2_ exerts its reliable and well-documented antioxidant effect by directly scavenging hydroxyl radicals, which represents the only fully confirmed and consensus-recognized mechanism of H_2_ at present. Whether H_2_ can regulate the epigenetic modification of biological macromolecules by modulating endogenous free radical levels remains worthy of in-depth exploration. All the above-mentioned antioxidant mechanisms are fundamentally based on the direct chemical reaction between H_2_ and reactive free radicals, especially hydroxyl radicals. Some studies have shown that iron porphyrin can act as an effective catalyst for H_2_-mediated catalytic hydrogenation reactions (Yao et al., 2022), while this catalytic mechanism still lacks sufficient in-depth verification and remains a preliminary research speculation. In addition, some scholars have proposed the hypothesis that H_2_ may act on protein molecules in a manner similar to inert gas binding (Turan et al., 2023); however, given the extremely small molecular size of H_2_, the possibility of H_2_ binding through a xenon-like protein pocket is relatively low and currently unsupported by solid experimental evidence. Recently, a study by Harvard University researchers published in Cell revealed for the first time that H_2_ in the gut is not an inert gas but rather serves as a key substrate or promoting factor for the enzyme 21-dehydroxylase, driving gut bacteria such as Eggerthella lenta to efficiently convert biliary glucocorticoids into progestins with neuroactive and sex hormone functions (e.g., allopregnanolone). This “enzymatic regulation hypothesis” proposes one potential specific molecular target for the physiological functions of H_2_ (McCurry et al., 2025; Yang et al., 2025). In 2026, Zhao et al. adopted multiple rigorous experimental techniques, including live-cell calcium imaging, *in vivo* two-photon imaging of small animals, gene-editing-based amino-acid mutation technology, transcriptome analysis, and molecular docking verification, and provided experimental evidence suggesting that H_2_ acts as a novel gaseous signaling molecule to regulate cellular calcium homeostasis via the TRPC4-TRPC4AP axis. The 730Arg-731Arg motif in the TRPC4 protein is identified as the core H_2_-sensitive site, which enables intracellular calcium ion homeostasis to maintain a dynamic balance and prevents excessive calcium accumulation in cells. Notably, this calcium regulatory mechanism is a newly proposed emerging model and has not formed academic consensus.

Nevertheless, the calcium influx triggered by H_2_ may potentially affect vascular tension and cellular electrophysiological stability, and this functional possibility requires further systematic validation in relevant disease models (Figure 5). In any case, the exploration and confirmation of the direct molecular action targets of H_2_ remains a core and challenging scientific issue to be addressed in future H_2_ medical research. In summary, only the hydroxyl radical-scavenging antioxidant effect of H_2_ is well-established, whereas epigenetic regulation, iron porphyrin-dependent catalysis, xenon-like protein binding, enzymatic progesterone regulation, and TRPC4-mediated calcium modulation are all emerging hypotheses without definitive consensus evidence.

**FIGURE 5:**
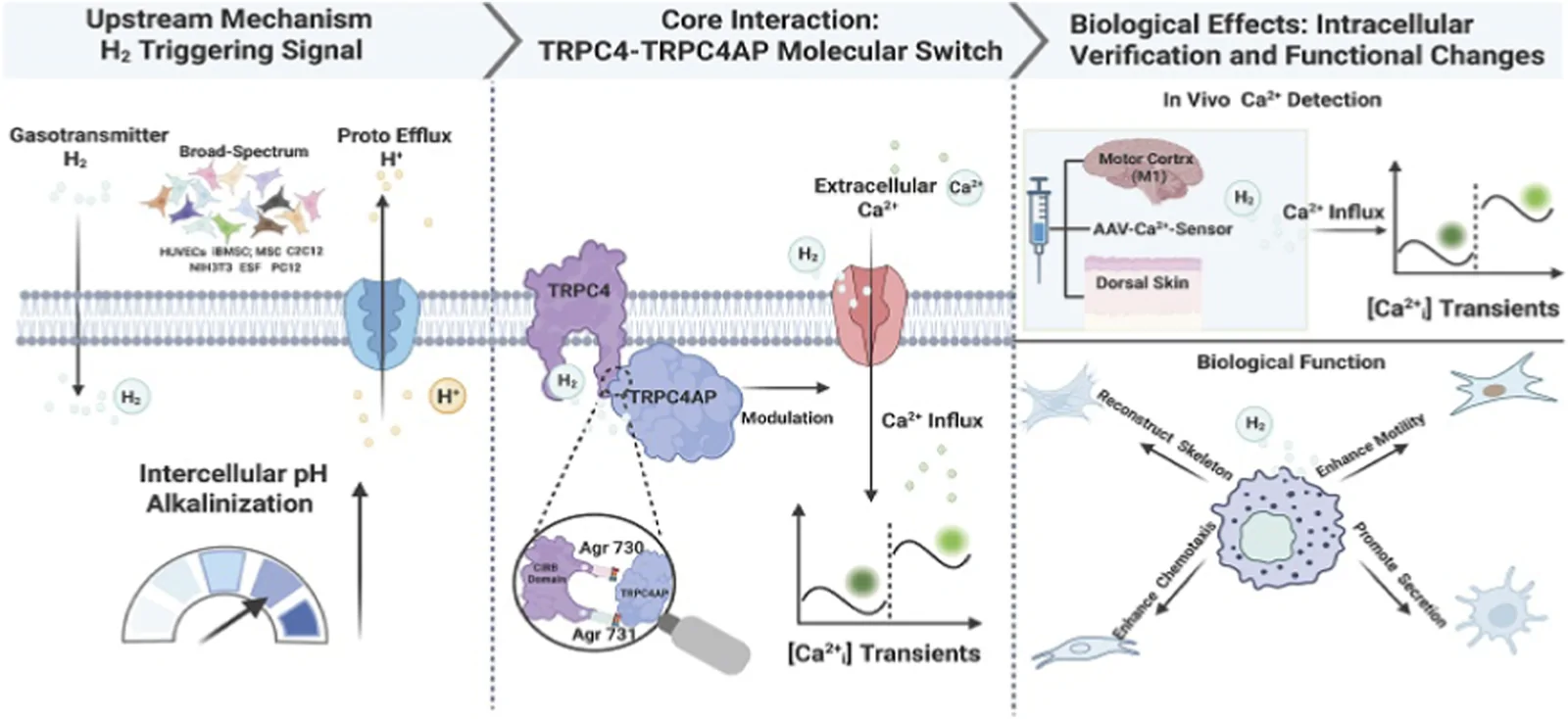
H_2_ as a novel gaseous signaling molecule that can regulate Ca^2+^ channels via the TRPC4-TRPC4AP axis (reprinted with permission from Zhao et al., 2026, licensed under CC BY 4.0).

## 5 H_2_ and tumors

A growing body of evidence supports the anti-tumor effects of H_2_, but the underlying mechanisms still need to be better understood to guide clinical use. H_2_ is known for its broad physiological actions, including antioxidant, anti-inflammatory, and anti-apoptotic effects. Accumulating studies indicate that its ability to selectively neutralize highly toxic ROS–mainly ·OH and ONOO^−^, which the body cannot efficiently clear–appears to be the core mechanism behind its anti-tumor activity (Zhou et al., 2024; Yang et al., 2020; Cao Y. et al., 2025). This selective property gives H_2_ an edge over common antioxidants like vitamins C and E (Ria et al., 2025; Russell, 2025). H_2_ also reduces ROS levels by boosting SOD and GSH while suppressing NOX2 expression (Jin et al., 2025; Zhao et al., 2019; Yang et al., 2020). NOX family enzymes are among the main sources of cellular ROS (Miyata et al., 2017). In urological cancers, NOX1, NOX2, NOX4, and NOX5 have been linked to tumor formation, cell growth, apoptosis, and blood vessel formation (Miyata et al., 2017). For instance, NOX4 drives angiogenesis through VEGF and HIF-1α, whereas NOX5 helps prostate cancer cells survive (Miyata et al., 2017). Oncogene activation and hypoxia push ROS production higher in cancer cells. These cells can tolerate moderately high ROS and even use them to fuel malignancy–ROS boost NF-κB, Akt/mTOR, and Wnt/β-catenin pathways, along with oncogenes like Ras, Bcr/Abl, and c-Myc, sustaining rapid metabolism and proliferation. At the same time, ROS-dependent signaling promotes invasion and metastasis. Yet when ROS levels become too high, they can eventually limit tumor progression; this is why non-selective antioxidant therapy might actually backfire (Zhou et al., 2024).

Several factors help explain the potential mechanisms by which H_2_ works against tumors. The highly cytotoxic radicals ·OH and ONOO^−^ can break DNA base pairs and double strands, and the body has no dedicated systems to remove them, leading to mitochondrial damage and excess ROS that raise cancer risk. Studies have shown that H_2_ specifically scavenges these radicals, reducing cellular damage and helping prevent cancer from starting. In the tumor microenvironment, high ROS levels boost NF-κB, Akt/mTOR, Wnt/β-catenin, and oncogenes such as Ras, Bcr/Abl, and c-Myc, promoting tumor cell growth, invasion, and spread; by clearing ROS from cancer cells, H_2_ may block these ROS-driven pathways and curbs cancer progression. H_2_ also works through immune cells: it protects the mitochondrial Na^+^/K^+^ ATPase, raises Bcl-2 levels, prevents the release of apoptotic factors like caspase-9, and keeps mitochondrial membranes intact, while lowering PD-1 expression on CD8^+^ T cells, reducing T cell exhaustion, and restoring normal levels of functional CD4^+^, CD8^+^ T cells, and NK cells (Meng et al., 2024; Dutta et al., 2026). Moreover, H_2_ directly regulates the Keap1-Nrf2-ARE and Nrf2-HO-1 pathways to maintain redox balance, markedly increasing antioxidant enzymes (SOD, GPx, CAT, HO-1) and boosting the cell’s ability to eliminate ROS (Xie et al., 2023; Hasegawa et al., 2022; Zhou et al., 2024; Dhillon et al., 2024; Mohd Noor et al., 2023). By clearing ROS, H_2_ eases the side effects of radiation and chemotherapy, improves appetite and overall health, and enhances quality of life in cancer patients (Mohd Noor et al., 2023). Beyond these, H_2_ has been reported to suppresses tumor growth, migration, and invasion, and triggers apoptosis. It lowers STAT3/Bcl2, ERK, AKT, Wnt, and VEGF, shifts macrophages toward the M1 phenotype while restraining M2, and induces pyroptosis via the ROS/NLRP3/caspase-1/GSDMD pathway. H_2_ also activates Nrf2/ARE, Keap1-Nrf2-ARE, and p38/MAPK, increases SOD/GPx/CAT/HO-1, lowers MPO and MMP-9, and stabilizes mitochondrial membrane potential (Yang et al., 2020; Zhou et al., 2024; Kura et al., 2022). Importantly, H_2_ protects normal cells–such as intestinal epithelium, bone marrow, heart, and liver–by raising Bcl-2/Bcl-xl and lowering Bax and caspases, while pushing cancer cells toward apoptosis; the mechanistic basis for this differential effect remains an emerging hypothesis (Yang et al., 2020). H_2_ also lessens the toxicity of chemotherapy (cisplatin, doxorubicin, 5-FU, cyclophosphamide) and radiotherapy without hurting their anti-tumor effects. Within the TME, H_2_ shields TIL mitochondria, cuts PD-1 expression, and restores T cell function through NADH/NADPH and PGC-1α. It enters mitochondria, neutralizes ROS, protects Na^+^/K^+^ ATPase, boosts Bcl-2, and blocks VDAC1 and caspase-9 (Mo et al., 2019). As for stage-specific effects–initiation, promotion, progression–the evidence is too weak to draw conclusions; we clearly flag this as a speculative area that needs future work. Overall, preclinical studies suggest that H_2_ may exert anti-tumor effects while potentially preserving anti-tumor immunity and reducing therapy-related side effects (Zhou et al., 2024; Yang et al., 2020) (Figure 6).

**FIGURE 6:**
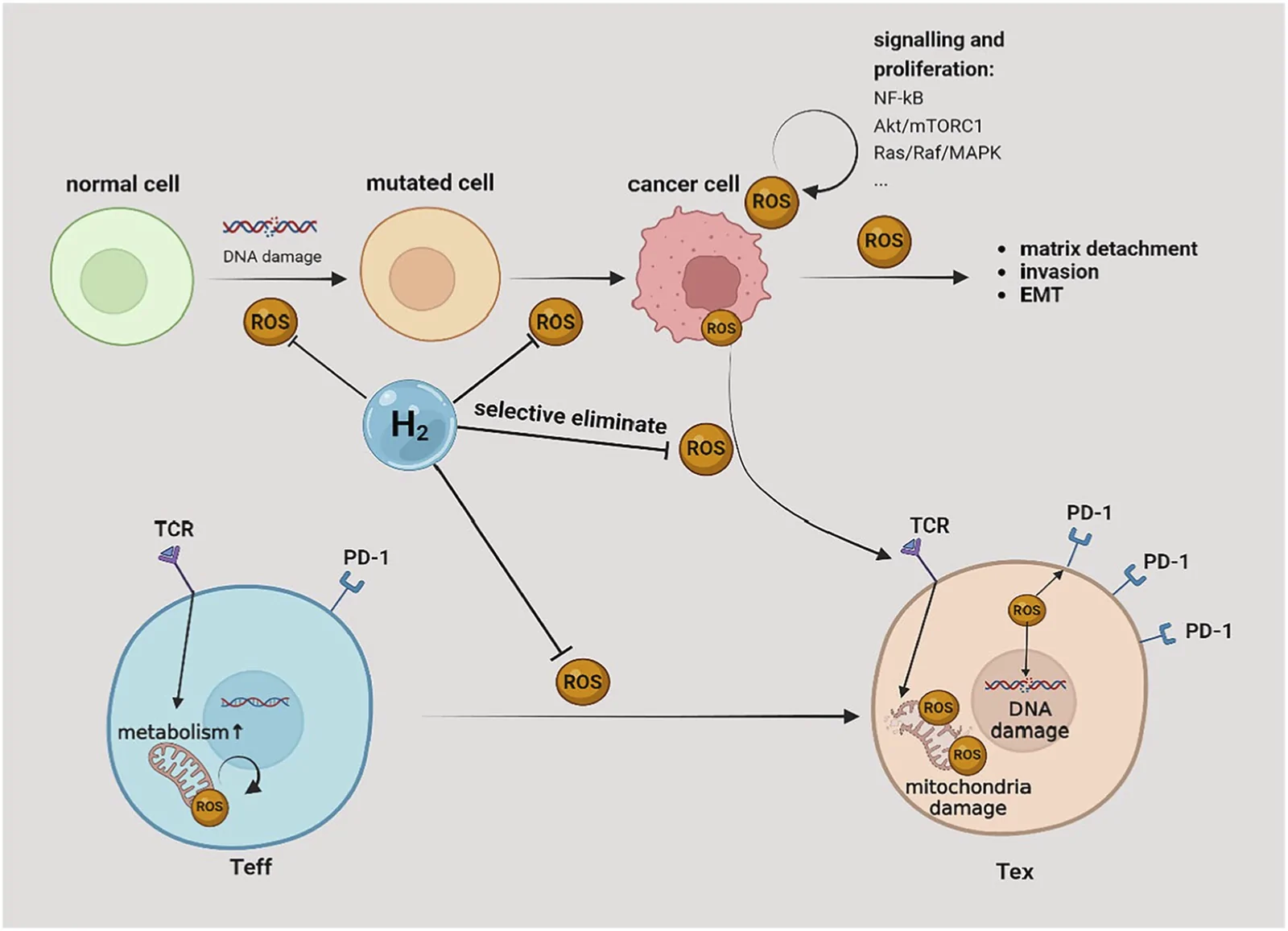
Anti-tumor activity of H_2_ achieved by selective elimination of ROS in the tumor microenvironment (reprinted with permission from Zhou et al., 2024, licensed under CC BY 4.0).

### 5.1 Anti-tumor effects of H_2_

H_2_ therapy has emerged as a preliminary candidate with limited clinical evidence for the adjunctive prevention and treatment of cancer, while standardized regimens, large-scale phase III trials and long-term safety data remain largely lacking. Oxidative stress is closely associated with the occurrence and development of malignant tumors. ROS can damage DNA, proteins, and cell-membrane lipids, serving as an inducement for cell carcinogenesis. Based on its selective antioxidant effect, H_2_ shows some preclinical evidence of efficacy in preventing tumorigenesis (Khiji et al., 2024; He et al., 2023; Li and Ding, 2025; Zheng et al., 2022; Nan et al., 2014). Critically, the existing antioxidant mechanism cannot fully explain H_2_’s anti-tumor activity. Most relevant mechanistic explorations belong to pilot basic studies instead of formal controlled clinical trials, and their translational reliability remains to be validated. However, the current antioxidant theory alone fails to adequately explain the anti-tumor mechanism of H_2_. In contrast, traditional antioxidants have a long-standing history of research on their effects on tumors, yet none of them can achieve satisfactory clinical outcomes. Therefore, the antioxidant mechanism of H_2_ in tumor treatment is not yet fully elucidated, and there may be other unknown mechanisms for hydrogen’s anti-cancer properties. H_2_ medicine research indicates that H_2_ can inhibit regulators of chromosome condensation (Li and Ai, 2017), certain key molecules in stem cells (Kawamura et al., 2018), proliferation (Kawamura et al., 2018), angiogenesis (Kawamura et al., 2018), etc., which may provide new perspectives for the research on hydrogen’s anti-tumor effects.

In 2019, a single-arm prospective follow-up study (Level 6 evidence) involving 82 patients with stage III and IV cancers who received HI therapy. The follow-up period ranged from 3 to 46 months. After 4 weeks of HI, patients reported subjective improvements in multiple clinical symptoms, including fatigue, insomnia, anorexia, and pain. Participants (41.5%) showed improved physical condition, with effects observed in patients with lung cancer. Among 58 patients with upregulated tumor markers, HI therapy led to a 36.2% reduction, with lung cancer patients experiencing the greatest decrease. It should be emphasized that alterations in tumor markers are merely biological changes and do not equate to definite clinical efficacy. In comparison, patients with pancreatic and liver cancers had more modest reductions. Imaging assessments of 80 patients with visible tumors showed an overall disease control rate of 57.5%. These findings hint at a tentative signal that HI therapy may help improve quality of life and partially control tumor progression in patients with advanced-stage cancers (Chen et al., 2019a).This non-randomized and uncontrolled study lacks a placebo group, so placebo effects and confounding variables cannot be excluded. Its moderate sample size also limits statistical robustness, generalizability and reproducibility.

Between 2014 and 2017, a non-randomized controlled clinical study (Level 3 evidence) in Japan analyzed 55 patients with stage IV colon cancer who received chemotherapy with the XELOX (CapeOX) regimen combined with HI therapy. The study examined the relationship between PD-1 expression on circulating CD8^+^ T cells and both progression-free survival (PFS) and overall survival. Results showed that H_2_ inhalation decreased the proportion of exhausted PD-1^+^ CD8^+^ T cells while increasing the number of active PD-1^-^ CD8^+^ T cells. As a result, patients experienced better PFS and overall survival, suggesting that H_2_ may modulate immune function to boost antitumor activity and improve prognosis (Akagi and Baba, 2019). These changes in immune biomarkers only reflect an associative correlation, and a reliable causal relationship between H_2_ intervention and improved clinical outcomes has not been confirmed. Unlike the observational trials mentioned above, this investigation combined H_2_ supplementation with standard chemotherapy as a preliminary controlled pilot trial, yielding higher-grade clinical evidence than single-arm observational studies. Owing to limited sample size and enrollment confined to a single geographic region, the trial omitted strict placebo and untreated control arms, making it impossible to isolate the inherent therapeutic effect of chemotherapy from spontaneous fluctuations in patients’ immune function. Furthermore, analyses were limited to peripheral CD8^+^ T-cell markers alone, with no complementary assessments of the tumor microenvironment or *in situ* immune features. As such, a causal link between H_2_ treatment and improved patient outcomes could not be established, and the study’s findings cannot be reliably reproduced across diverse cancer cohorts.

Several case reports further demonstrate the potential of H_2_ therapy in cancer treatment. A patient with recurrent gallbladder cancer received daily HI therapy. Although tumor progression continued during the first month, tumor size and tumor marker levels gradually declined, eventually normalizing. Such changes in tumor indicators cannot be regarded as conclusive evidence of therapeutic effects. Approximately two and a half months after starting treatment, the patient resumed normal daily activities and was alive 10 months later. This phenomenon, called pseudo-progressive remission, resembles the clinical pattern observed in tumors treated with PD-1 checkpoint inhibitors (Chen et al., 2019b). In another case, a 72-year-old woman with bile duct cancer experienced progressive reduction of intra-abdominal metastatic lesions after 3 months of HI therapy. Her anemia and hypoproteinemia were corrected, lymphocyte counts and tumor marker levels normalized, and she regained the ability to perform daily activities independently (Chen et al., 2019c). These are case reports (Level 6 evidence), representing low-level clinical evidence. Without control or placebo design, they have poor statistical power and reproducibility, and multiple confounding variables cannot be ruled out.

In 2019, a small-sample single-arm study (Level 6 evidence) with 20 patients suffering from advanced non-small cell lung cancer (NSCLC) examined the effects of 2 weeks of HI therapy on immune function. Results showed notable improvements in both the quantity and functionality of immune cells. Although these immune enhancements did not necessarily correlate with a reduction in tumor size, they appeared to modulate the immunosuppressive state and change the tumor microenvironment (TME), possibly reducing tumor aggressiveness (Chen et al., 2020a),The limitations of this paper are the small number of participants and the short duration of H_2_ treatment, so the following issues remain unknown: whether prolonged HI can continue to improve immune function, what the optimal level of immune improvement is, whether the therapeutic effects rebound after cessation of HI, and whether improving immune function before treatment reduces tumor recurrence and metastasis. These questions should be further investigated in future research (Chen et al., 2020a). Similarly, a 44-year-old woman with lung cancer and multiple metastatic lesions underwent 3 months of H_2_ therapy. During this period, intra-abdominal metastatic spots gradually decreased in size, anemia and hypoproteinemia were corrected, and lymphocyte counts and tumor marker levels returned to normal levels. The patient was able to resume normal daily activities, indicating the potential of HI therapy to improve overall clinical condition (Chen et al., 2019d). This is an individual case report (Level 6 evidence) with highly limited reproducibility. In 2020, a controlled clinical study (Level 3 evidence) involving 58 patients with advanced NSCLC reported that H_2_ therapy alleviated pulmonary symptoms and extended PFS compared to the control group. Among all patients receiving combined H_2_ therapy, PFS was markedly longer than in controls. Most drug-related adverse events observed in the combined therapy group gradually decreased or disappeared entirely. These findings suggest that HI therapy appears to potentially assist in slowing tumor progression and may mitigate adverse effects associated with traditional anticancer treatments (Chen et al., 2020b). The limited sample size and incomplete reporting of randomization details affect statistical robustness. Multi-center trials are required to confirm its reproducibility. Due to its simplicity, low cost, and minimal adverse reactions, H_2_ inhalation is a favorable clinical approach for the rehabilitation and supportive care of patients with advanced cancers, meriting further research in larger, controlled trials. Moreover, the antioxidation mechanism of H_2_ is only one aspect of its effects, and there may be other modes of action. The research on the direct and indirect signaling pathways mediated by H_2_ is still in the exploratory stage. Whether H_2_ has a broad-spectrum anti-tumor effect or is effective against specific tumors needs further determination. The top priority in H_2_ anti-tumor research is to discover the exact mechanism of hydrogen’s anti-tumor effect. More basic and clinical experiments are required to further confirm the role of H_2_ and clarify its mechanism of action at the molecular level. The possibility of publication bias (overrepresentation of positive results) should also be considered.

### 5.2 H_2_ alleviate radiotherapy-induced adverse effects

Patients undergoing radiotherapy frequently experience fatigue and a reduction in quality of life (QoL), which are often attributed to increased OS and inflammatory responses induced by ROS generated during treatment (Li et al., 2025). In a randomized, placebo-controlled study (Level 2 evidence), Kang et al. (Kang et al., 2011) examined the effects of HRW in 49 patients with malignant liver tumors receiving radiotherapy. The study demonstrated that consumption of HRW for 6 weeks considerably lowered circulating reactive oxygen metabolites and maintained the overall oxidative potential of the blood. Patients who received HRW reported higher QoL scores during radiotherapy than the control group. Despite rigorous trial design, the small sample size and lack of long-term data restrict its reproducibility; potential confounding variables from combined treatment also exist. The C4 Clinic in Tokyo, Japan, studied whether H_2_ therapy could alleviate bone marrow suppression in cancer patients undergoing radiotherapy. Between 2015 and 2016, 23 patients with advanced cancer participated in the study. The results showed that HI markedly attenuated reductions in white blood cells (WBCs) and platelets, reducing bone marrow damage caused by intensity-modulated radiation therapy (IMRT) without compromising the anti-tumor efficacy of radiotherapy (Hirano et al., 2021). This small-sample single-arm study (Level 6 evidence) has no placebo or control group, so placebo effects and confounding factors cannot be excluded. Further validation is needed to improve result reproducibility. These findings suggest H_2_ may exert antioxidant effects by decreasing ROS production and reducing inflammation, which could help mitigate radiotherapy-induced adverse reactions. This highlights the potential of H_2_ as a supportive treatment to improve tolerance and QoL for cancer patients receiving radiotherapy (Table 1).

**TABLE 1:** Effect of H_2_ intervention in cancer clinical trials.

Disease type	Sample size	Routes of H_2_ delivery	Duration	Main effects	Authors and year
Non-small cell lung cancer	20	HI	2 weeks	Exhausted cytotoxic T cells and senescent cytotoxic T cells↓, total natural killer T cells, Killer Vδ1 and Vδ2 cells↑	Chen et al. (2020a)
Non-small cell lung cancer	58	HI	5 months	The prevalence of symptoms↓ Progression-free survival↑	Chen et al. (2020b)
Advanced cancer	82	HI	4 weeks	Tumor markers ↓, quality-of-life and control cancer progression↑	Chen et al. (2019b)
Gallbladder cancer	1	HI	10 months	The tumor size and tumor markers ↓ CD8+PD-1+ T cells↓	Chen et al. (2019c)
Lung cancer with multiple metastases	1	HI	12 months	All brain tumors had disappeared and no significant changes in metastases in the liver and lung	Chen et al. (2019d)
Colorectal cancer	55	HI	Triennium	PD-1- CD8^+^ T cells↑, PD-1+ CD8^+^ T cells↓ progression-free survival↑	Akagi and Baba (2019)
Radiotherapy for tumors	49	Drinking HRW	6 weeks	Reactive oxygen metabolites↓ QOL scores ↑	Kang et al. (2011)
Radiotherapy for tumors	23	HI	4 weeks	IMRT-induced bone marrow damage↓ white blood cells and platelets ↓	Hirano et al. (2021)
Liver function of cancer with chemotherap	144	Drinking HRW	66 months	ALT,AST and IBIL↓	Yang et al. (2017)

### 5.3 Synergistic and hypotoxic effects of H_2_ on chemotherapy

Chemotherapy is a cornerstone in the treatment of malignant tumors; however, its lack of tumor specificity often results in considerable adverse effects, including cardiotoxicity, bone marrow suppression, oral mucositis, and gastrointestinal inflammation (Shi et al., 2018). By leveraging its antioxidant properties, H_2_ has been studied for its ability to enhance the efficacy of chemotherapy while reducing its toxicity (Table 1). A randomized, single-blind controlled trial (Level 2 evidence) involving 144 patients with colon cancer evaluated the effects of HRW during standardized mFOLFOX6 chemotherapy. Patients who consumed HRW showed no prominent changes in liver function before and after treatment, while the placebo group had higher levels of alanine aminotransferase, aspartate aminotransferase, and indirect bilirubin. These findings suggest that H_2_ provides hepatoprotective effects during chemotherapy (Yang et al., 2017). This trial has a relatively sufficient sample size, but it only focuses on liver function. Long-term efficacy and comprehensive toxicity outcomes are missing, requiring more translational and clinical verification. Moreover, H_2_ appears to possibly potentiate chemotherapy outcomes while alleviating treatment-related toxicity, which suggests tentative potential for it to act as an adjunct intervention that may enhance patient tolerance and therapeutic responses during cancer treatment.

Research on the anti-tumor effects of H_2_ has grown progressively in recent years (Yang et al., 2022; Zhang et al., 2022; Mohd Noor et al., 2023; Yin et al., 2025). H_2_ exerts antioxidant activity via scavenging hydroxyl radicals. This mechanism was initially demonstrated in landmark studies published in Science (Dole et al., 1975), and Nature Medicine (Ohsawa et al., 2007), and is currently acknowledged as a core biological mechanism of H_2_. However, hydroxyl radical scavenging alone cannot fully explain the diverse biological functions of H_2_; its core molecular targets, key signaling pathways, and disease-specific regulatory mechanisms remain unclear. Accordingly, elucidating the precise anti-tumor mechanisms of H_2_ is a key priority of current research. Additional basic and clinical studies are required to further validate the biological effects of H_2_ and clarify its underlying molecular mechanisms. The antioxidant property of H_2_ represents only one of its functional pathways, suggesting the existence of alternative mechanisms of action. Studies investigating H_2_-regulated direct and indirect signaling pathways are still in the preliminary stage, and it remains unconfirmed whether H_2_ exerts broad-spectrum anti-tumor activity or targeted effects on specific tumor subtypes. Furthermore, tumor heterogeneity imposes new requirements for H_2_ delivery strategies and serves as a critical factor limiting its therapeutic efficacy. Therefore, developing tumor pathology-adapted H_2_ delivery systems and characterizing the dose–effect relationship of H_2_ are important directions for future research. Clinical trials of H_2_ therapy are far less abundant than preclinical investigations. Existing studies also exhibit inconsistent sample sizes, and most focus on respiratory and gastrointestinal cancers, resulting in insufficient data for other tumor types. Additionally, the majority of relevant studies are conducted in East Asian populations, which may limit the generalizability of the reported therapeutic outcomes to other populations. Future research should include more *in vivo* experiments and clinical investigations to further evaluate the safety, efficacy, and long-term impacts of H_2_ therapy for cancer treatment. In summary, H_2_ shows promising potential for tumor prevention and treatment, which warrants further systematic research.

## 6 H_2_ and metabolism-related diseases

The antioxidant effect of H_2_ has been confirmed in various metabolic disease models (Xie et al., 2023). However, the selective ·OH scavenging activity of H_2_ cannot explain its extensive beneficial effects. From the current research, H_2_ may have multiple targets of action. H_2_ may exert its anti-oxidant effect through three mechanisms, namely, direct free radical scavenging, inhibition of ROS generation, and enhancement of anti-oxidant enzyme activity (Xie et al., 2023) (Figure 7). Future research should focus on identifying the potential targets of H_2_ and exploring how H_2_ interacts with target molecules to induce cellular responses and subsequently produce beneficial effects on metabolic diseases.

**FIGURE 7:**
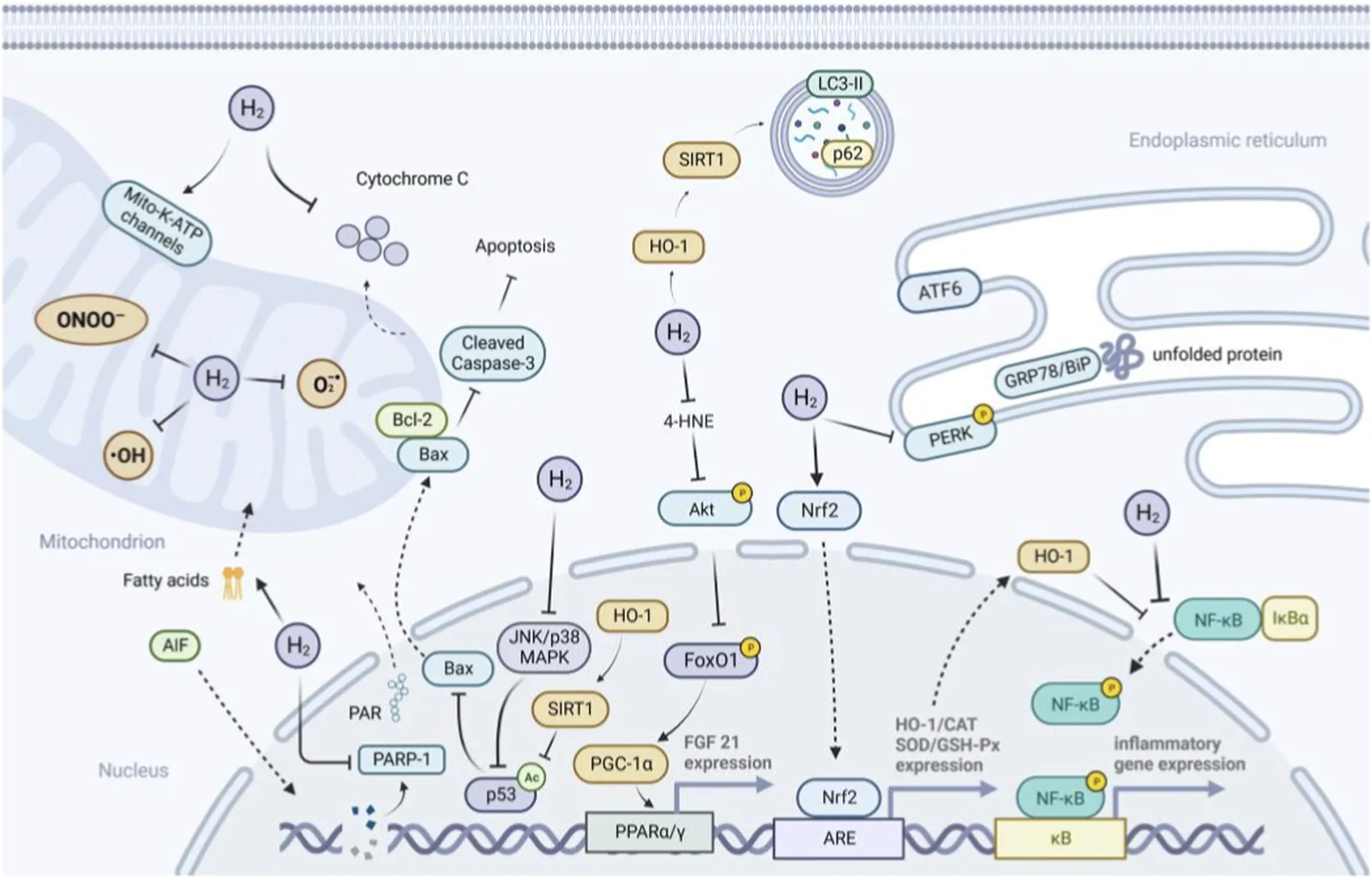
The possible mechanisms underlying the effects of H_2_ on metabolic diseases (reprinted with permission from Xie et al., 2023, licensed under CC BY 4.0).

### 6.1 H_2_ and hyperuricemia

Hyperuricemia is a metabolic disorder arising from an imbalance in purine metabolism, leading to elevated serum uric acid levels. Oxidative stress induced by free radicals has been identified as a key factor in developing metabolic disorders, including hyperuricemia (Cui et al., 2025). A randomized, double-blind placebo-controlled trial (Level 2 evidence) examined the effects of HRW in 100 male patients diagnosed with hyperuricemia. Participants were arranged into three groups: placebo (n = 32), low-dose HRW (n = 35), and high-dose HRW (n = 33). The placebo group consumed three bottles of normal drinking water daily; the low-dose group consumed two bottles of HRW and one bottle of normal water; and the high-dose group consumed three bottles of HRW for 8 weeks. The results showed that the high-dose HRW regimen was more effective at lowering serum uric acid levels than the low-dose regimen. After 8 weeks of treatment, the high-dose HRW group had a notable reduction in uric acid levels from baseline (Wu et al., 2024). These results suggest that H_2_ warrants further investigation as a potential therapeutic option for managing hyperuricemia (Table 2). This high-quality RCT sets rigorous trial design with placebo control, but it only recruits male patients, bringing gender-related confounding variables. The single-center design limits its generalizability and reproducibility for female patients.

**TABLE 2:** Clinical effects of H_2_ intervention in metabolically related disorders.

Disease type	Sample size	Routes of H_2_ delivery	Duration	Main effects	Authors and year
Participants	100	Drinking HRW	8 weeks	Blood uric acid ↓, alkaline phosphatase ↓	Wu et al. (2024)
T2DM	30	Drinking HRW	8 weeks	LDL ↓, urinary 8-isoprostanes ↓, Free fatty acids ↓, adiponectin ↑ Extracellular-superoxide dismutase ↑	Kajiyama et al. (2008)
T2DM	50	Drinking HRW	3 months	Serum lactate concentration ↓	Ogawa et al. (2021)
Diabetes	9	Drinking HRW	8 weeks	Urinary 8-hydroxy-2-deoxyguanosine ↓ Blood glucose ↓, fructosamine ↓, 1,5-anhydro-D-glucitol ↑	Asada et al. (2020)
Obesity	10	Drinking HRW	4 weeks	Body fat percentage ↓, arm fat index ↓, Fasting serum insulin levels ↓, Serum triglycerides ↓	Korovljev et al. (2018)
Obesity	36	Drinking HRW	8 weeks	Mitigated cravings ↓, sleep quality ↑ Total cholesterol ↓, LDL cholesterol ↓, Plasma glucagon-like peptide-1 levels ↑	Todorovic et al. (2025)
Healthy females	20	HI	7 months	Respiratory exchange ratio ↓, Ventilation across all intervals↓, body fat percentage↓	Grepl et al. (2025)
NAFLD	12	Drinking HRW	28 days	Serum aspartate transaminase levels↓, Liver fat content ↓	Korovljev et al. (2019)
NAFLD	30	Drinking HRW	8 weeks	Lactate dehydrogenase levels ↓, Body mass index ↓, NF-κB ↓, MMP-9 ↓, Heat shock protein 70 ↓	Kura et al. (2022)
NAFLD	43	HI	13 weeks	LDL-C ↓, AST ↓, ALT↓, MDA ↓,SOD ↑ TNF-α↓, IL-6↓	Tao et al. (2022)
MetS	20	Drinking HRW	8 weeks	SOD ↑, TBARS↓, HDL ↑ TC/HDL-C↓	Nakao et al. (2010)
MetS	60	Drinking HRW	24 weeks	Serum hemoglobin A1c ↓, Blood cholesterol ↓, glucose levels ↓	LeBaron et al. (2020)
MetS	20	Drinking HRW	10 weeks	HDL ↑, LDL-C↓,TC↓, TNF-α↓, SOD ↑ Apo B100 ↓, apo E↓	Song et al. (2013)

### 6.2 H_2_ and diabetes

Diabetes is characterized by increased OS, driven by excessive ROS production. This oxidative imbalance induces lipid, protein, and DNA peroxidation, which play a key role in the development of diabetes and its related complications (Reza et al., 2021). HRW has been studied for its potential to reduce OS and enhance metabolic parameters in diabetic patients. Kajiyama et al. (2008) conducted a randomized, double-blind, placebo-controlled crossover trial (Level 2 evidence) involving 30 patients with type 2 diabetes mellitus (T2DM) managed through diet and exercise, along with six participants with impaired glucose tolerance. After an 8-week HRW intervention, considerable decreases in low-density lipoprotein and eight-isoprostane levels were observed, along with improvements in glucose tolerance. The limited sample size reduces statistical robustness, and the crossover design may cause residual intervention confounding effects. Further evidence was provided by a multicenter, prospective, randomized, double-blind study involving 50 patients with T2DM. Participants were assigned to the HRW and control groups. While insulin resistance (IR) did not differ considerably between groups, the HRW cohort showed a notable decrease in lactate levels. This reduction was strongly correlated with improvements in fasting glucose, fasting insulin, and IR, suggesting that H_2_ may have beneficial effects on glucose homeostasis by modulating oxidative and metabolic pathways (Ogawa et al., 2021). Asada et al. (2020) administered 1,500 mL of HRW daily for 8 weeks to nine patients with abnormal diabetes-related serum markers. This is a small-sample single-arm study (Level 6 evidence) without placebo or control group, so placebo effects and daily dietary confounding variables cannot be excluded, with poor result reproducibility. Post-intervention analyses revealed notable reductions in fasting blood glucose, increases in 1,5-anhydro-D-glucitol content and production rates, and decreases in urinary 8-hydroxy-2′-deoxyguanosine levels and production rates, reflecting systemic alleviation of DNA oxidative damage. These clinical studies suggest H_2_ intake may help attenuate OS, lower peroxidative damage and ameliorate several biochemical markers in diabetic patients. By modulating oxidative pathways, HRW can slow disease progression and reduce the risk of diabetes-related complications, suggesting a possible adjunctive role that requires further confirmation in metabolic disorders.

### 6.3 H_2_ and obesity-related diseases

Obesity is a multifaceted metabolic condition characterized by excessive adipose tissue accumulation, along with endocrine dysregulation, persistent low-grade inflammation, and increased OS. Its complex etiology makes long-term management challenging. Korovljev et al. (2018) evaluated the effects of HRW in a double-blind, placebo-controlled crossover trial (Level 2 evidence) involving ten overweight, middle-aged women. The extremely small cohort size reduces statistical effectiveness, and the single-female-subject setting restricts result generalizability. Participants consumed HRW for 4 weeks, while the control group received H_2_-free mineral water. Post-intervention analysis revealed significant reductions in total body fat percentage and arm fat mass in the HRW group. A remarkable decrease in serum triglycerides was also observed, while other lipid fractions remained unchanged. Fasting insulin concentrations decreased by 5.4% after HRW administration, in contrast to a 29.3% rise observed in the placebo phase. These results suggest that H_2_ intake may improve metabolic health in people with obesity, likely due to its antioxidant and anti-inflammatory effects. A randomized, double-blind, placebo-controlled study (Level 2 evidence) conducted in 2025 examined the metabolic effects of an 8-week HRW regimen in 36 people with obesity. HRW intake led to reduced appetite, better subjective sleep quality, lower total cholesterol and low-density lipoprotein cholesterol, and increased circulating glucagon-like peptide-1 levels. No severe adverse events occurred during the study (Todorovic et al., 2025). Another crossover study investigated the immediate physiological effects of HI. A total of 20 healthy women inhaled H_2_ via nasal cannula for 60 min, and their respiratory gas measurements were compared with those in a placebo condition. This crossover study adopts healthy participants rather than obese patients, which cannot support therapeutic conclusions for obese populations, with limited translational reliability. HI dramatically decreased the respiratory exchange ratio (RER) and minute oxygen consumption. The extent of RER decrease was positively correlated with body fat percentage, suggesting that H_2_ might affect substrate utilization patterns (Grepl et al., 2025). These studies indicate that HRW and H_2_ inhalation may be safe adjunctive approaches for appetite control and for improving metabolic markers in people with obesity. Long-term H_2_ exposure may contribute to weight reduction and improved metabolic health (Table 2).

### 6.4 H_2_ and non-alcoholic fatty liver disease

Non-alcoholic fatty liver disease (NAFLD) has emerged as the most prevalent liver disorder globally, yet effective treatment options are still limited. Considering that metabolic dysregulation is a key contributor to NAFLD pathogenesis, therapeutic agents that can improve lipid and glucose metabolism, like H_2_, have potential for disease management (Korovljev et al., 2019). Korovljev et al. (2019) conducted a double-blind, placebo-controlled, crossover trial (Level 2 evidence) involving 12 patients with severe NAFLD. The small sample cohort may cause statistical bias, and short 28-day intervention cannot verify long-term curative effect. Participants were randomly assigned to receive either 1 L of HRW or placebo water daily for 28 days. The study showed that liver fat deposition in the H_2_-treated group was lower than in the placebo group, with liver fat content decreasing from 284.0 ± 118.1 mmol to 256.5 ± 108.3 mmol. Serum aspartate aminotransferase levels declined by 10.0% after the 28-day H_2_ intervention. In another randomized placebo-controlled study (Level 2 evidence), 30 NAFLD patients consumed HRW daily for 8 weeks. The results showed positive trends in weight loss and BMI among the HRW group. Improvements were also noted in lipid profiles and lactate dehydrogenase levels. H_2_ therapy was associated with substantial reductions in NF-κB, heat shock protein 70, and matrix metalloproteinase-9 (MMP-9) levels (Kura et al., 2022). Tao et al. (2022) further examined the effects of H_2_ inhalation in a 13-week randomized, placebo-controlled trial (Level 2 evidence) involving 43 participants. The study showed that H_2_ inhalation improved serum lipid profiles and liver enzyme activity. Moreover, imaging assessments, including ultrasound and computed tomography, demonstrated a substantial reduction in liver fat content in patients with moderate-to-severe NAFLD. These clinical studies demonstrate preliminary trends that H_2_ therapy, whether administered via HRW or inhalation, has beneficial effects on hepatic fat accumulation, metabolic indicators, and inflammatory markers. These results support the potential of H_2_ as an additional treatment option for managing NAFLD. Most included NAFLD trials are single-center studies, and unified H_2_ intervention dosage is lacking, which reduces cross-study reproducibility.

### 6.5 H_2_ and metabolic syndrome

Oxidative stress is recognized as a key factor in the development and progression of metabolic syndrome (MetS) (Bharaj et al., 2025). Clinical studies have shown that H_2_ can positively impact metabolic health and oxidative balance in patients with MetS. One study involved 20 individuals with MetS who consumed HRW daily for 8 weeks. This is a small-sample single-arm study (Level 6 evidence) without placebo or control group, so placebo effects and daily dietary confounding variables cannot be excluded, with poor result reproducibility. After the intervention, SOD activity increased by 39%, while urinary thiobarbituric acid reactive substances decreased by 43%. HDL-C levels rose by 8%, and the total cholesterol (TC) to HDL-C ratio decreased by 13% (Nakao et al., 2010). LeBaron et al. (2020) further examined the effects of H_2_ in a randomized, placebo-controlled trial (Level 2 evidence) involving 60 participants with MetS. Participants were assigned to either the HRW group or the placebo group for 24 weeks. The HRW group showed substantial reductions in fasting blood TC and glucose levels, along with decreases in serum glycated Hb. Improvements were also observed in biomarkers of inflammation and OS, highlighting H_2_’s role in enhancing redox homeostasis. Modest reductions in BMI and waist-to-hip ratio suggested a potential effect on adiposity-related risk factors. In another study, 20 participants with potential metabolic syndrome consumed HRW daily for 10 weeks. This is a small-sample observational study (Level 6 evidence) with undefined diagnostic criteria for potential MetS, which limits result credibility and clinical extrapolation. The intervention led to reductions in serum TC and LDL-C, as well as substantial decreases in Apolipoprotein B100 (apoB100) and apoE. Furthermore, OS markers were substantially reduced, underscoring the antioxidative properties of H_2_ (Song et al., 2013). These findings indicate that H_2_ supplementation, particularly via HRW, can reduce OS, improve lipid and glucose metabolism, and regulate inflammatory responses. Therefore, H_2_ may serve as a tentative adjunct strategy that warrants additional exploration for the potential prevention and management of metabolic syndrome.

Collectively, available preclinical and clinical data indicate that H_2_ intervention may confer beneficial effects on metabolic diseases. In line with the evidence-based medicine hierarchy, existing studies encompass high-level randomized controlled trials, moderate-quality controlled studies, low-grade single-arm observational investigations, and preliminary preclinical pilot work. These studies exhibit graded evidentiary strength rather than uniform clinical credibility. Nonetheless, therapeutic responses vary across individual studies, and several investigations have noted additional biological outcomes. Variations in hydrogen concentration, treatment duration, H_2_ intervention timing, and disease severity may account for these inconsistent results (Xie et al., 2023). Current clinical observations suggest the potential utility of H_2_ in metabolic disease management, although high-quality clinical evidence remains limited. Most existing studies are constrained by small sample sizes, single-center designs, unclear blinding strategies, and unavoidable confounding factors. Some trials cannot exclude placebo effects, accompanied by limited statistical reliability and inconsistent reproducibility across different populations. Large-scale, randomized, controlled, multicenter, double-blind, long-term intervention studies are required before the routine clinical use of hydrogen therapy can be considered. With ongoing progress in clinical and mechanistic research, H_2_ therapy may serve as an evidence-based adjuvant approach for the management of various metabolic disorders. However, standardized treatment protocols, large-scale phase III trials, and long-term safety data are currently unavailable. The mechanisms underlying hydrogen’s biological actions remain incompletely clarified and represent a key research challenge. While several hypotheses have been proposed to explain hydrogen’s bioactivity, most studies attribute its function primarily to antioxidant regulation. Free radicals exert context-dependent biological roles in disease progression. Even hydroxyl radicals, the most cytotoxic reactive oxygen species, do not exclusively induce cellular damage. For example, superoxide anions and their downstream hydroxyl radicals are critical for bacterial clearance by human phagocytes. Therefore, hydrogen’s selective hydroxyl radical scavenging effect alone cannot fully explain its broad biological activities. Current evidence indicates that H_2_ may modulate multiple molecular targets, and the biological phenotypes induced by H_2_ intervention may result from the integrated interactions between H_2_ and its target molecules. Future mechanistic studies are warranted to identify key molecular targets of H_2_ and characterize the regulatory mechanisms by which hydrogen-target interactions modulate cellular behaviors. In general, existing research supports the potential applicability of H_2_ in the clinical management of metabolic diseases.

## 7 H_2_ and nervous system diseases

### 7.1 H_2_ and neurodegenerative diseases

Oxidative stress plays a crucial role in the development of neurodegenerative disorders, with increased damage in individuals carrying the apolipoprotein E4 (APOE4) allele dramatically contributing to disease onset (Jofre-Monseny et al., 2008). H_2_, recognized for its cytoprotective properties, has emerged as a potential treatment and preventive strategy for mild cognitive impairment (MCI) (Yuan et al., 2023). Nishimaki et al. (2018) conducted a randomized, double-blind, placebo-controlled trial (Level 2 evidence) involving 73 MCI patients who consumed 300 mL of HRW daily, while the control group received regular drinking water. Over a 1-year intervention, no overall differences in total AD Assessment Scale–Cognitive Subscale (ADAS-Cog) scores were observed between groups; however, APOE4 carriers in the HRW group showed considerable improvements in both the total ADAS-Cog score and the word recall subtest, indicating a genotype-specific benefit. This study adopts stratified analysis based on APOE4 genotype, but the *post hoc* subgroup analysis lacks preset trial endpoints, which may increase false-positive statistical risk; besides, it only focuses on cognitive scales without detecting neural oxidative stress indicators. In a separate study with eight AD patients, inhaling 2% H_2_ gas provided symptom relief and slowed disease progression. This is an extremely small-sample single-arm observational study (Level 6 evidence), without placebo control and parallel control group, placebo effect and daily nursing confounding factors cannot be excluded, with low clinical evidence credibility. Diffusion tensor imaging (DTI) analyses over a 6-month follow-up revealed improvements in neuronal integrity, which were maintained at the 1-year follow-up (Ono et al., 2023). Parkinson’s disease (PD), a neurodegenerative disorder strongly associated with aging (Zhang et al., 2025b; Lin et al., 2022), has also been studied for H_2_-based treatments. In a mouse model of PD, sustained H_2_ preconditioning for approximately 7 days exerts prominent neuroprotective effects. Mechanistically, orally administered H_2_ dissolved in drinking water triggers gastric ghrelin synthesis and secretion by activating β1-adrenergic receptors. Circulating ghrelin subsequently crosses the blood-brain barrier and binds to its functional receptor, growth hormone secretagogue receptor, thereby mediating neuroprotection. Consistently, H_2_ treatment upregulates ghrelin mRNA expression in ghrelin-secreting SG-1 cells. Collectively, these findings elucidate the chronic biological effects of H_2_ and highlight the favorable potential of H_2_-based strategies for the prevention and treatment of PD (Noda et al., 2019).

In a randomized controlled trial (Level 2 evidence) (Yoritaka et al., 2013), 48 PD patients who had previously been treated with levodopa were randomized to consume either 1 L of HRW daily or regular water for 48 weeks. This trial failed to implement double-blind design, and background levodopa dosage was not unified among enrolled patients, bringing obvious medication confounding bias. Evaluations using the Unified PD Rating Scale (UPDRS) showed worsening of motor and non-motor symptoms in the control group, while the HRW group exhibited symptomatic improvement. These findings tentatively suggest H_2_ may slow neurodegenerative progression by reducing OS and preserving neuronal function, suggesting a possible role that requires further study for the management of MCI, AD, and PD.

### 7.2 H_2_ and cerebral infarction

Experimental research has shown that HI provides neuroprotective benefits in models of cerebral infarction (Yuan et al., 2023). The above conclusion is derived from *in vivo* animal model experiments (Preclinical evidence), which cannot completely simulate complex clinical complications and combined medication status of stroke patients. Based on these preclinical observations, clinical studies evaluated its therapeutic potential in human stroke. A randomized controlled trial (Level 2 evidence) involving 50 patients with mild to moderate acute cerebral infarction found that HI treatment led to considerable clinical improvements. The trial included only mild-moderate stroke patients, excluding severe cerebral infarction cases, resulting in population selection bias; intervention concentration and inhalation time of H_2_ were not standardized. Patients receiving H_2_ showed clear reductions in magnetic resonance imaging (MRI) relative signal intensity, along with improvements in National Institutes of Health Stroke Scale (NIHSS) scores and physical therapy assessments (Ono et al., 2017). These findings provide preliminary hints that H_2_ could be an adjunctive approach that warrants further investigation for treating acute cerebral infarction. Although the exact mechanisms are not yet fully understood, current hypotheses suggest that they involve reducing post-ischemic inflammation and inhibiting excessive ROS formation. Both processes are known to contribute to secondary neuronal damage after ischemic events. More mechanistic studies are needed to explore the molecular pathways through which H_2_ mediates neuroprotection (Table 3).

**TABLE 3:** The effect of H_2_ intervention in nervous and skin system diseases clinical trials.

Disease type	Sample size	Routes of H_2_ delivery	Duration	Main effects	Authors and year
MCI	73	Drinking HRW	12 months	APOE4↓,Word recall task score↑ Total ADAS-cog score↑	Nishimaki et al. (2018)
AD	8	HI	6 months	ADAS-cog score↑, neuronal integrity↑, disease progression↓	Ono et al. (2023)
PD	17	Drinking HRW	48 weeks	UPDRS scores↑	Yoritaka et al. (2013)
Cerebral infarction	50	HI	2 weeks	NIHSS score↓, Physical therapy assessment↓	Ono et al. (2017)
Sleep disorders	66	HI	7 days	Pittsburgh Sleep quality index ↓, self-rating depression Scale↓	Gao et al. (2026)
Psoriasis	41	HRW bathing	8 weeks	PASI score↓, Pruritus↓	Wu et al. (2024)
Psoriasis	3	HI Drinking HRW, Drop infusion of HRS	5 days	DAS28 system↓, PASI score↓, TNF-α↓, IL-6↓, IL-17↓,	Ishibashi et al. (2015)
Healthy volunteers	6	HRW bathing	3 months	The wrinkles at the back of the neck↓	Kato et al. (2012)
PU	22	Drinking HRW	4 months	Wound size↓	Li et al. (2013)
Acne	24	Hydrogen purification	14 days	Skin eruptions↓, Sebum↓ Skin hydration↑	Chilicka et al. (2022)
Acute erythematous skin diseases	4	Drop infusion of HRS, HI	3 days	Erythema ↓, skin ulcers ↓, edematous hardening ↓, Intensive pain in the face↓, Acute fever↓,nausea ↓, headache↓,	Ono et al. (2012)
PV	2	Drinking HRW Apply wet compress with HRW	10 weeks	Infection↓, Anabrosis↓,	Yang et al. (2019)

### 7.3 H_2_ and sleep disorders

Sleep disturbances are closely linked to multiple chronic health conditions, including cardiovascular and cerebrovascular diseases, as well as neurodegenerative disorders like AD (Yan M. et al., 2024). Due to its anti-inflammatory, antioxidant, and anti-fibrotic effects, H_2_ has been suggested as a possible treatment to improve sleep quality and manage sleep pressure–related behavioral responses (Vincent et al., 2023). A single-blind, randomized controlled clinical study (Level 2 evidence) examined the effectiveness of inhaling an H_2_–oxygen gas mixture in individuals with sleep disorders. Only single-blind design was adopted, participants could distinguish intervention gas, which cannot eliminate subjective reporting bias; the short 7-day intervention cycle cannot verify long-term sleep improvement effect. Approximately 66 participants were assigned to either a control group or an H_2_–oxygen inhalation group. Those in the intervention group received nasal inhalation therapy for seven consecutive days. Sleep-related metrics in the control group remained largely unchanged, while participants inhaling H_2_–oxygen showed improvements. Total sleep duration and sleep efficiency increased considerably on days 3, 5, and 7, while wakefulness after sleep onset decreased dramatically on days 3 and 7. After 1 week of treatment, participants receiving hydrogen–oxygen therapy showed lower scores on the Pittsburgh Sleep Quality Index and Self-Rating Depression Scale compared to controls. However, no substantial differences were observed for the Self-Rating Anxiety Scale (Gao et al., 2026). These results suggest that H_2_-based gas therapy may enhance the sleep cycle, decrease wakefulness, and alleviate psychological burden, but current evidence is limited.

With its small molecular size and high permeability, H_2_ can readily diffuse and function at the subcellular level, including neuronal mitochondria, cell nuclei and other substructures. Due to its selective antioxidant properties, H_2_ may alleviate the pathological progression of multiple neurological disorders, such as neurodegenerative diseases, cerebral ischemia–reperfusion injury, atherosclerosis, and post-traumatic neuroinflammatory responses. Although preliminary small-scale clinical trials have suggested the therapeutic potential of H_2_ in PD and AD, large-scale clinical studies have not yet yielded consistent positive outcomes. Such inconsistencies may be related to variations in intervention timing, duration, administration modality, and H_2_ concentration. Furthermore, the underlying molecular mechanisms and applicable scope of H_2_ therapy remain incompletely clarified. Further basic and clinical investigations are therefore warranted to verify the biosafety, optimal dosage, appropriate intervention timepoints and delivery strategies of H_2_, thereby supporting the development of novel therapeutic approaches for neurological diseases. Despite preliminary preclinical and clinical observations, the clinical translation of H_2_ therapy may be more complex than that of conventional targeted drugs designed for specific disease indications.

## 8 H_2_ and skin-related diseases

H_2_ may directly interact with potent oxidants in the body, such as hydroxyl radicals. Hydroxyl radicals may play physiological roles by initiating free radical chain reactions and influencing the regulation of calcium or ATP-dependent potassium channels in mitochondria (Guo and Zhang, 2025; Ohta, 2021). Calcium ions (Ca^2+^) and their concentration gradient in the epidermis are crucial for regulating key skin functions, such as keratinocyte differentiation, skin barrier formation, and maintenance of the osmotic barrier. Recent studies have shown that intracellular Ca^2+^ stores, especially those in the endoplasmic reticulum (ER), are important contributors to the epidermal calcium gradient. Appropriate ER calcium balance is essential for processes such as keratinocyte differentiation, intercellular junction development, antibacterial defense, and maintenance of the osmotic barrier. H_2_ inhibits lipid peroxidation caused by free radicals and restores calcium ion levels. This restoration of calcium ion levels subsequently regulates calcium-dependent gene expression (Zhao et al., 2026; Iuchi et al., 2016). H_2_ also upregulates the expression of genes regulated by H3K27 methylation, such as Kcnc3, in various organs. H_2_ also changes the methylation state of H3K27 by inducing the H3K27 demethylase Jmjd3 and activates genes related to the mitochondrial unfolded protein response (mtUPR) (Guo and Zhang, 2025; Sobue et al., 2017). These findings suggest that H_2_ promotes beneficial effects by activating mtUPR, which is driven by epigenetic histone modifications and changes in gene expression.

### 8.1 H_2_ and psoriasis

Psoriasis and plaque-type psoriatic lesions are chronic, immune-mediated inflammatory skin diseases that often resist available treatments. H_2_-based therapies have recently gained attention as possible adjuncts for modulating inflammatory pathways in these conditions. A randomized, controlled pilot study (Level 2 evidence) evaluated the therapeutic effects of HRW bathing in 41 patients with psoriasis over 8 weeks. This pilot trial has no double-blind design, and cutaneous lesion scoring relies on subjective clinician evaluation, bringing assessment bias. Participants assigned to HRW bathing showed marked improvements in Psoriasis Area and Severity Index (PASI) scores compared to controls, along with reductions in pruritus intensity. In patients with plaque-type psoriatic arthritis, 33.3% achieved complete resolution of skin lesions, while 66.7% showed partial remission by week eight (Zhu et al., 2018). Further insights were reported by Ishibashi, who explored multiple H_2_ delivery methods, including intravenous HRS, HI, and oral HRW, in three patients with psoriasis complicated by psoriatic arthritis. This is an extremely small-sample observational study (Level 6 evidence), without unified intervention dosage and parallel control group. Regardless of the method used, patients experienced decreases in Disease Activity Score-28 (DAS28) and PASI values. Moreover, circulating pro-inflammatory cytokines, such as TNF-α, IL-6, and IL-17, decreased during H_2_ treatment (Ishibashi et al., 2015). These clinical observations point toward H_2_’s anti-inflammatory potential,potential, but further evidence is needed before considering it a treatment option for chronic inflammatory skin disorders, particularly psoriasis.

### 8.2 H_2_ and skin wrinkles

Prolonged exposure to ultraviolet (UV) radiation induces substantial oxidative damage in skin fibroblasts, leading to signs of photoaging such as wrinkle formation, skin laxity, telangiectasia, and pigmentation changes (Teng et al., 2025). In a preliminary study, Kato et al. (2012) evaluated the effects of bathing in HRW on skin wrinkle severity. Six healthy adults participated in a 3-month treatment of daily HRW baths. This is a small-sample single-arm preliminary study (Level 6 evidence), lacking placebo bathing control, and daily sun protection behavior acts as uncontrolled confounding factor. Four of these participants showed visible reductions in wrinkle depth on the dorsal and cervical areas after the treatments. These results hint at that long-term exposure to HRW might reduce skin OS and help prevent UV-related wrinkle development.

### 8.3 H_2_ and pressure ulcers

Pressure ulcers (PU) frequently occur in older adults with limited mobility or in patients with neuromuscular disorders, including spinal cord injury, amyotrophic lateral sclerosis, multiple sclerosis, and muscular dystrophy (Bani Assad and Sadri, 2025). Li et al. (2013) conducted an observational study on 22 elderly hospitalized patients with severe PU, all of whom experienced eating difficulties and had bedsores due to complex underlying illnesses. This is a non-controlled observational study (Level 6 evidence), which cannot exclude the natural self-healing effect of chronic wounds. Normal wound care was provided, and each patient received 600 mL of HRW daily through a feeding tube as part of their fluid intake. markedly, adding HRW to enteral hydration was associated with remarkable reductions in wound size and accelerated healing in these critically ill individuals. This result suggests that HRW delivered via feeding tube may serve as a beneficial supportive therapy for PU management in admitted patients.

### 8.4 H_2_ and acne

Acne can have a considerable psychological impact, leading to decreased self-esteem, depression, and even suicidal thoughts in affected individuals (Lin et al., 2025). A randomized controlled clinical study (Level 2 evidence) involving 24 women with mild acne and increased sebum production evaluated the therapeutic efficacy of H_2_-based skin cleansing alone or combined with cosmetic acids. This trial only enrolled female participants, resulting in gender selection bias; limited sample size weakens statistical reliability of combined therapy efficacy. Participants were assigned to two treatment protocols: Group A received H_2_ purification with the H_2_ Jet device, which delivers pressurized alkaline H_2_ water, while Group B was treated with H_2_ purification supplemented with a 40% formulation containing phytic, pyruvic, lactic, and ferulic acids. Both groups completed four sessions at 14-day intervals. Skin condition was evaluated at baseline and again 30 days after the final session. H_2_-based therapy decreased acne lesions and improved overall skin tone and texture. The combination of H_2_ purification with cosmetic acids produced better results compared to monotherapy. H_2_ treatment did not induce irritation, erythema, or other adverse dermatologic effects (Chilicka et al., 2022). These findings point to the potential of H_2_-based interventions may be safe and warrant further study for managing acne and enhancing overall skin health.

### 8.5 H_2_ and acute erythematous skin diseases

Acute erythematous skin disorders often necessitate urgent medical attention, particularly when they occur with severe pain or fever. In such emergencies, clinicians may need to start treatment promptly before identifying the underlying etiology or evaluating the functional status of other organ systems. Erythema typically results from inflammation that causes skin redness, which can be the primary pathological mechanism or a secondary effect (Robert and Kupper, 1999). Ono et al. (2012) documented four clinical cases of acute erythematous skin disease in which patients presented with rashes and severe associated symptoms, including intense pain, fever, neurological deficits, skin ulceration, generalized edema, sclerosis, nausea, and headache. This is a small case-series observation (Level 6 evidence), with no blank control group, and curative effect cannot be separated from delayed efficacy of routine medication. All patients initially received standard pharmacological therapy; however, this did not result in adequate improvement. Following this, H_2_ therapy was administered as an adjunctive intervention. The outcomes showed a clear resolution of erythema and substantial improvement in associated symptoms across all four cases. Features such as severe facial pain with neurological impairment, febrile erythema with edematous sclerosis, acute febrile episodes along with nausea and headache, and progressive erythematous lesions with flushing exhibited substantial improvement after H_2_ therapy, with no recurrence reported during follow-up. These findings suggest that H_2_ may be a potential therapeutic option for acute erythematous dermatoses resistant to standard care.

### 8.6 H_2_ and pemphigus vulgaris

Pemphigus vulgaris (PV) is a chronic, immune-mediated blistering disease. Despite advances in immunosuppressive treatments, PV continues to be associated with considerable morbidity and a mortality rate nearly three times higher than that of the general population (Gorini et al., 2025). In 2019, Yang et al. (2019) reported the first clinical use of HRW wet-compress therapy to treat PV. This is a two-case retrospective observation (Level 6 evidence), lacking controlled comparison, and perioperative nursing confounding factors cannot be eliminated. The initial case involved a 62-year-old woman presenting with erythema on the right tibial area and a 5 × 8 cm ulcer with purulent discharge and hemorrhagic crusts. Microbiological tests showed mixed infection with *Citrobacter* koseri, Alternaria, and *Staphylococcus aureus*. After a 6-week course of systemic antibiotics, the patient developed antibiotic-induced hepatotoxicity, requiring the discontinuation of antimicrobial therapy. Three weeks after stopping antibiotics, cultures continued to show intermittent fungal and bacterial presence. The patient started HRW wet-compress therapy given locally twice daily for 1 h, without any additional antimicrobial agents. After 1 month, microscopic examination and microbiological cultures turned negative, and complete ulcer healing occurred within 10 weeks. A second case involved a male patient in his fifties, also diagnosed with PV, who received an identical HRW wet-compress protocol. His ulcerative lesions similarly showed progressive improvement and complete resolution within 10 weeks. These observations indicate that HRW wet-compress therapy merits further investigation for chronic inflammatory skin ulcers, particularly in cases complicated by infection and resistant to traditional therapies.

H_2_ possesses a low molecular weight and strong transdermal permeability. It can scavenge cutaneous hydroxyl radicals, stabilize epidermal calcium homeostasis, and modulate histone epigenetic modifications and mitochondrial stress responses, thereby regulating skin inflammation, keratinocyte proliferation, and wound repair progression. Current clinical studies have illustrated the protective and therapeutic effects of H_2_ intervention via multiple administration routes, including H_2_ bathing, topical wet compresses, inhalation, and skin cleansing. Although available data show preliminary positive results, relevant research remains in the early stage, with variable therapeutic efficacy observed across different administration approaches and dosages. Furthermore, existing skin-related studies present several limitations, including non-standard local hydrogen intervention parameters, inadequate blinding design, limited sample sizes, and insufficient long-term follow-up data. Therefore, further investigations are essential to advance H_2_ research in dermatological diseases. Expanded research scopes and larger sample cohorts are required for clinical studies, while in-depth mechanistic exploration is needed to elucidate the regulatory effects of H_2_ on gene expression and cellular signaling during skin regeneration, which may improve the understanding of its therapeutic value. For personalized clinical application of H_2_ therapy, it is necessary to optimize H_2_ delivery strategies and refine dose–effect criteria, including standardized intervention dosage, treatment timing, intervention duration, and post-treatment efficacy evaluation. Additionally, the combination of H_2_ therapy with conventional dermatological treatments such as antioxidants and retinoids may improve therapeutic outcomes, providing valuable references for future translational studies.

## 9 H_2_ and respiratory system diseases

Respiratory disorders pose a major global health burden and are the third leading cause of death worldwide. Despite extensive research, effective therapeutic options are still limited, and the demand for new interventions persists. OS-induced cellular senescence has been identified as a key pathogenic mechanism in chronic respiratory diseases, especially chronic obstructive pulmonary disease (COPD) and idiopathic pulmonary fibrosis. It contributes substantially to disease progression (Liu et al., 2025). A 2020 clinical study assessed the short-term effects of inhaling a vapor mixture containing 2.4% H_2_ (XEN) in 10 patients with asthma and 10 with COPD. This is a small-sample observational study (Level 6 evidence) without blinding design, and unified baseline medication of enrolled patients was not controlled. Participants inhaled the gas mixture for 45 min, after which investigators observed multiple inflammatory biomarkers, i.e., granulocyte–macrophage colony-stimulating factor, IFN-γ, IL-1β, IL-2, IL-4, and IL-6, in both peripheral blood and exhaled breath condensate (EBC). Results showed decreased monocyte chemoattractant protein-1 (MCP-1) levels in both asthma and COPD groups, along with lower IL-8 levels in the asthma group. Soluble CD40 ligand levels increased in the EBC of COPD patients, while IL-4 and IL-6 concentrations decreased markedly in both groups. These changes reveal that HI can reduce airway inflammation in patients with asthma and COPD (Wang S. T. et al., 2020). In 2023, a prospective, randomized, double-blind clinical trial (Level 2 evidence) investigated the therapeutic effect of an H_2_–oxygen gas mixture in individuals with acute exacerbations of COPD (AECOPD) who had a Breathlessness, Cough, and Sputum Scale (BCSS) score ≥6. This high-quality trial adopts strict inclusion criteria, but it only evaluates short-term symptom improvement without detecting long-term pulmonary function changes. Patients were randomly assigned to receive either the H_2_–oxygen mixture or standard oxygen therapy. Between Day 2 and Day 6, the reduction in BCSS scores was dramatically higher in the H_2_–oxygen group. Moreover, cough assessment scores remained consistently lower than those of controls, indicating that H_2_-containing therapy provides symptomatic relief in COPD (Zheng et al., 2021). A 2025 randomized, parallel-group comparative study (Level 2 evidence) further examined combination gas therapies in 100 post-COVID-19 patients hospitalized with acute COPD exacerbations. This trial fails to set unified gas inhalation duration, and patients received irregular auxiliary ventilation treatment, bringing intervention confounding bias. Participants had documented SARS-CoV-2 pneumonia before admission. The trial compared the safety and efficacy of combined helium–oxygen (t-He/O_2_), nitric oxide (NO), and H_2_ therapy with single-gas treatments. Results showed that the combination regimen, administered alongside standard etiological treatment and non-invasive ventilation, was more effective and had a better safety profile compared with monotherapy approaches (Shogenova. 2025). Another clinical study assessed the effectiveness of HI in 35 patients with severe acute tracheal stenosis. This is a single-arm uncontrolled study (Level 6 evidence), lacking conventional treatment control group to verify exclusive H2 efficacy. Inhalation of a hydrogen–oxygen gas mixture markedly reduced inspiratory airflow resistance, and the intervention was considered safe and well tolerated (Zhou et al., 2019). These results suggest that further investigation is warranted for H_2_-based inhalation strategies in various respiratory conditions. HI may represent an adjunctive approach that requires confirmation in larger trials for managing airway inflammation, improving respiratory function, and relieving symptoms in both acute and chronic respiratory diseases (Table 4).

**TABLE 4:** The effect of H_2_ intervention in respiratory system diseases clinical trials.

Disease type	Sample size	Routes of H_2_ delivery	Duration	Main effects	Authors and year
Asthma	10	HI	1 day	Monocyte chemotactic protein 1↓, IL-8↓, IL-4 and IL-6 levels in EBC↓	Wang X. et al. (2020)
COPD	10	HI	1 day	Monocyte chemotactic protein 1↓, EBC soluble cluster of differentiation-40 ligand↑, IL-4 and IL-6 levels in EBC↓	Wang S. T. et al. (2020)
AECOPD	108	HI	7 days	Cough assessment test score↓, BCSS score↓	Zheng et al. (2021)
COPD	100	HI	14 days	Signs of hypoxemia and hypercapnia↓, Vascular endothelial dysfunction ↓, metabolic disorders↓, the shunt fraction↓ oxygen delivery to tissues↑	Asada et al. (2020)
Tracheal stenosis	35	HI	1 day	EMGdi↓, Pdi↓, borg score↓, Resistance parameters↓	Zhou et al. (2019)
COVID-19	90	HI	3 days	Chest distress ↓, chest pain↓, Cough Scale↓, Resting oxygen saturation↑	Guan et al. (2020)
COVID-19	24	HI	50 days	Neutrophil percentage↓, C-reactive protein↓	Luo et al. (2022)
COVID-19	50	HI	14 days	6 MWT↑, FVC↑, FEV1↓	Botek et al. (2022)

Patients with COVID-19 often show acute symptoms, including headache, fever, dry cough, difficulty breathing, and extreme fatigue. After the initial illness phase, some patients develop post-acute sequelae, commonly called post-COVID-19 syndrome, characterized by persistent symptoms such as severe fatigue, dyspnea, headache, and cognitive difficulties, which may persist beyond 4 weeks after initial symptom onset. The cause may be partly attributed to their inability to restore redox and inflammatory balance, and the antioxidant and anti-inflammatory effects of H_2_ have certain potential in addressing this issue (Alwazeer et al., 2021). A pioneering multicenter clinical study (Level 2 evidence) conducted across seven hospitals in China enrolled 100 COVID-19 patients to assess the efficacy of HI therapy.The trial adopts open-label design without blinding, and patients received differentiated basic supportive treatment. Compared with conventional oxygen therapy, continuous HI administration over 2 days dramatically reduced disease severity and improved clinical parameters, including dyspnea, cough, chest tightness, chest pain, and arterial oxygen saturation (Guan et al., 2020). Further supporting evidence comes from a 2022 retrospective analysis (Level 6 evidence) of medical records from 12 COVID-19 patients who received inhalation of an H_2_-oxygen gas mixture. Retrospective data collection exists information bias, and sample size is too small for statistical inference.The study reported an evident reduction in the neutrophil ratio and a substantial decrease in serum CRP levels, indicating that HI effectively attenuates inflammatory responses (Luo et al., 2022). In the same year, another randomized controlled study (Level 2 evidence) examined the effects of low-flow HI in 50 patients recovering from acute COVID-19. This study only focuses on short-term rehabilitation indicators, without long-term follow-up for persistent post-COVID symptoms. The findings showed substantial improvements in functional exercise capacity, measured by the 6-min walk test (6MWT), along with gains in pulmonary function parameters, including forced vital capacity (FVC) and forced expiratory volume in one second (FEV1) (Botek et al., 2022). These studies manifest the therapeutic potential of HI in managing COVID-19, underscoring its ability to reduce inflammation, improve respiratory function, and enhance physical performance. These findings emphasize the clinical importance of H_2_ as an adjunctive strategy for both acute treatment and post-acute recovery in COVID-19 patients, but confirmatory trials are needed.

As a small-molecule gas with alveolar permeability, H_2_ can enter pulmonary tissue rapidly, eliminate excessive pulmonary ROS, alleviate airway and lung parenchymal inflammation, inhibit pulmonary fibrosis progression, and regulate systemic redox balance. Existing respiratory-related studies contain Level 2 double-blind RCTs, Level 3 imperfect controlled trials and Level 6 small-sample or retrospective studies. Current limitations include diversified H_2_ inhalation concentration and duration, incomplete blinding design, inconsistent basic medication schemes, and insufficient long-term outcome observation. Further standardized multicenter double-blind trials are required to optimize inhalation intervention protocols and validate the clinical application value of H_2_ for acute and chronic respiratory disorders. Despite the diverse advantageous properties of H_2_, its practical implementation in clinical and domestic scenarios remains limited. Safety concerns serve as the primary constraint, as hydrogen gas may become explosive when its atmospheric concentration exceeds 4%–5%. In addition, precise quantitative dosing of H_2_ presents notable technical difficulties. Furthermore, HRW exhibits insufficient stability, which acts as a key practical barrier. Existing research indicates that open systems lose approximately 2%–5% of dissolved H_2_ every 3 min (Zajac et al., 2025). In this context, the development of cost-effective and high-efficiency H_2_ capture and storage systems is necessary to mitigate H_2_ dissipation. Despite these challenges, H_2_ shows potential translational value for the prevention and management of respiratory diseases.

## 10 H_2_ and arthropathy

### 10.1 H_2_ and exercise fatigue and injury

Physical activity during training and competitive events imposes more physiological demands on athletes, often leading to various levels of fatigue. As a result, finding strategies that effectively reduce fatigue and improve recovery after exercise has become a key focus in sports science research (Freeman et al., 2025; Mu et al., 2026; Katada et al., 2015). Several clinical studies have examined the potential role of H_2_ as an intervention to combat exercise-induced OS, inflammation, and skeletal muscle fatigue. A controlled clinical trial (Level 3 evidence) with 20 participants (10 male and 10 female) showed that HI could improve exercise performance measures. This trial adopts open-label design without blinding, and individual exercise baseline ability brings uncontrollable confounding bias. HI increased peak running velocity by approximately 4%. Furthermore, after seven consecutive days of HI, participants experienced a 2% extension in time-to-exhaustion during endurance tests, along with a marked decrease in circulating insulin-like growth factor-1 (IGF-1) levels (Freeman et al., 2025). These results suggest that HI may modulate exercise physiology by attenuating metabolic stress and improving endurance capacity. In 2024, a randomized, placebo-controlled, double-blind, crossover study (Level 2 evidence) assessed the effects of H_2_ inhalation in 24 elite male rugby players. This high-quality trial only recruits male professional athletes, limiting the extrapolation of conclusions to female athletes and ordinary exercisers. Participants HI before high-intensity training sessions over 3 weeks. Compared to the placebo group, athletes receiving HI showed dramatically higher levels of nitric oxide, L-arginine, and tetrahydrobiopterin, molecules crucial for endothelial function and vascular regulation. Meanwhile, biomarkers of oxidative DNA damage, such as hydroxydeoxyuridine, and inflammatory mediators, including IL-6, were lower in the H_2_ group. Similarly, total antioxidant capacity was substantially higher in the HI-treated athletes (Zhao et al., 2024). These findings suggest that H_2_ provides both antioxidant and anti-inflammatory effects, ultimately lowering biochemical indicators of exercise-induced tissue stress and promoting better physiological recovery in professional athletes. Another study (Level 2 evidence), using a double-blind, randomized, balanced crossover design, examined the effect of H_2_ inhalation on fatigue resistance in 24 healthy adult males. All enrolled participants are young males, lacking subjects of different genders and age groups, with limited population applicability. The experimental group received 60 min of HI before performing a cycling ergometer fatigue test, while the control group inhaled regular atmospheric air. HI markedly improved several subjective and objective measures, including the visual analog scale (VAS) for fatigue, the rating of perceived exertion (RPE), cycling cadence during the last 30 s of the fatigue protocol, ·OH scavenging capacity, and post-exercise serum lactate levels (Dong et al., 2024). These findings suggest the potential of pre-exercise HI as an effective strategy for reducing fatigue and sustaining athletic performance during intense activity.

In addition to inhalation methods, extensive research has focused on oral consumption of HRW. A large-scale study (Level 2 evidence) evaluating HRW consumption before ergometer cycling exercise involved 99 untrained individuals and 60 trained participants. Unified daily water intake and diet were not strictly controlled, which may interfere with exercise metabolism indicators. HRW notably enhanced endurance performance and reduced psychological fatigue compared to placebo controls (Mikami et al., 2019). Exercise-induced acidosis, which often contributes to muscle discomfort and impaired performance, has also been examined in relation to HRW. In a randomized, double-blind, placebo-controlled study (Level 2 evidence) with 52 healthy male volunteers, 14 days of HRW ingestion led to a marked elevation in fasting arterial bicarbonate levels, indicating that HRW may help buffer metabolic acidosis during high-intensity exercise (Ostojic and Stojanovic, 2014). Furthermore, a 2-week regimen combining local and oral H_2_ administration was more effective than standard therapy in aiding recovery from soft-tissue injuries (Ostojic et al., 2014). Other research studies also support these findings. For example, eight male volunteers who consumed HRW before and after exhaustive exercise experienced reduced ROS production (small-sample single-arm study, Level 6 evidence, low statistical persuasiveness) (Dobashi et al., 2020). Similarly, a trial involving eight trained cyclists revealed that saturated HRW notably enhanced performance during intermittent cycling sessions lasting more than 30 min (small-sample observational study, Level 6 evidence) (Da Ponte et al., 2018). These clinical studies highlight the potential of both inhaled H_2_ and HRW as supportive interventions to improve athletic performance, accelerate post-exercise recovery, and reduce fatigue. Oral HRW, in particular, warrants further investigation as a possible rehydration and recovery aid for athletes involved in high-intensity or prolonged physical activities (Table 5).

**TABLE 5:** The effect of H_2_ intervention in arthropathy diseases clinical trials.

Disease type	Sample size	Routes of H_2_ delivery	Duration	Main effects	Authors and year
Healthy volunteers	20	HI	7 days	Peak running velocity↑, IGF-1↓	Freeman et al. (2025)
Rugby players	24	HI	3 weeks	NO↑, L-arginine↑, Tetrahydrobiopterin↑, Tydroxydeoxyguanosine↓, IL-6↓	Zhao et al. (2024)
Healthy volunteers	24	HI	1 h	VAS↑, RPE↑, hydroxyl radicals↓, Serum lactate↓	Dong et al. (2024)
Healthy volunteers	159	HI	1 day	Endurance↑, fatigue ↓	Mikami et al. (2019)
Healthy volunteers	52	Drinking HRW	14 days	Blood pH↑, fasting bicarbonates↑	Ostojic and Stojanovic (2014)
Professional athletes	36	Drinking HRW, topical H_2_ packs	14 days	Plasma viscosity↓, joint recovery↑	Ostojic et al. (2014)
Healthy volunteers	8	Drinking HRW	3 days	Accumulative muscular fatigue↓, Biological antioxidant potential	Dobashi et al. (2020)
Professional athletes	8	Drinking HRW	2 weeks	Peak power output↑	Da Ponte et al. (2018)
RA	20	Drinking HRW	4 weeks	DAS28↓, urinary 8-OHdG↓, C-reactive protein level↓	Ishibashi et al. (2012)

### 10.2 H_2_ and rheumatoid arthritis

Rheumatoid arthritis (RA) is a chronic autoimmune disorder recognized by persistent inflammation and progressive destruction of cartilage and bone. Although various anti-inflammatory medications are currently available, their long-term use is often restricted due to serious side effects, i.e., immunosuppression, which increases susceptibility to infections (McInnes and Schett, 2011; Mao et al., 2023; Budlewski et al., 2025). Therefore, discovering treatment strategies that effectively reduce inflammation without compromising immune function is critical for managing chronic inflammatory conditions. In this regard, H_2_ has emerged as a prospective candidate due to its strong anti-inflammatory properties and few side effects. Ishibashi et al. (2012) conducted a single-arm clinical study (Level 6 evidence) with 20 RA patients who first completed a 4-week washout period, followed by an 8-week intervention with HRW. This study has no parallel conventional treatment control group, and individual basic immunosuppressant dosage was not unified, existing intervention confounding factors. After the intervention, urinary levels of 8-hydroxydeoxyguanosine (8-OHdG), a marker of oxidative DNA damage, dropped by 14.3%, and systemic inflammation, measured by C-reactive protein (CRP) levels, was also dramatically reduced. Moreover, participants reported significant improvements in RA-related clinical symptoms. These findings imply that H_2_ can reduce OS and inflammatory responses in RA patients, suggesting a possible therapeutic effect that requires confirmation in larger trials. The ability of H_2_ to target chronic inflammation without inducing substantial adverse effects makes it a candidate for further study as an adjunct or alternative therapy to traditional anti-inflammatory treatments for RA.

H_2_ can eliminate joint excess ROS, inhibit synovial inflammatory infiltration, relieve exercise-induced skeletal muscle oxidative damage, stabilize immune homeostasis, and delay articular cartilage degeneration, thus exerting protective effects on musculoskeletal tissues. Current arthropathy-related studies cover Level 2 standardized double-blind crossover RCTs, Level 3 non-blinded controlled trials and Level 6 small-sample single-arm trials. Common research limitations include single gender enrollment, non-unified H_2_ intake dosage and intervention timing, lack of long-term follow-up on joint function, and undifferentiated physical exercise intensity. More multicenter, stratified controlled trials are required to optimize individualized H_2_ intervention schemes for sports injury rehabilitation and autoimmune joint diseases.

Studies regarding inflammatory bone and joint disorders have shown that H_2_ can suppress inflammatory responses, attenuate bone resorption and promote bone formation. Nevertheless, the mechanisms by which H_2_ regulates bone regeneration, tissue repair and bone mineralization remain incompletely understood. H_2_ may affect stem cell activity by modulating signaling pathways involved in stem cell proliferation and differentiation, thereby modulating osteogenesis and maintaining normal bone tissue metabolism (Yuan et al., 2023; Zhang et al., 2018). Although current data suggest that hydrogen may improve bone and joint conditions by regulating stem cell behaviors, its detailed mechanisms in these diseases still require further exploration. Targeting pro-inflammatory cytokines is a major therapeutic approach for inflammatory bone and joint diseases, yet this strategy often fails to achieve sustained remission, and disease recurrence is frequently observed after withdrawal of cytokine-targeted treatment. Reducing pro-inflammatory cytokine levels is one of the functional mechanisms of H_2_. However, H_2_ monotherapy may not be sufficient to achieve optimal therapeutic outcomes. Whether combined treatment with hydrogen and conventional agents such as non-steroidal anti-inflammatory drugs can improve therapeutic responses, extend treatment duration benefits and improve clinical prognosis remains to be further investigated. A growing body of evidence indicates that H_2_ exhibits anti-inflammatory, antioxidant and anti-apoptotic activities with few adverse reactions, making H_2_ biology a developing area in basic medical research. Further studies focusing on the precise mechanisms, key molecular targets and signaling pathways of H_2_ in bone and joint disorders will help provide reliable scientific support for its potential clinical application.

## 11 H_2_ and other diseases

### 11.1 H_2_ and gastroesophageal reflux disease

Gastroesophageal reflux disease (GERD) is a common clinical condition identified by the backward flow of gastric and duodenal contents into the esophagus. Growing evidence suggests that OS noticeably contributes to the development of GERD. Proton pump inhibitors (PPIs) remain the primary first-line treatment for managing this disorder (Sandhu and Fass, 2018; Jiang et al., 2025). In a controlled clinical study (Level 3 evidence), Franceschelli et al. (2018) evaluated 84 GERD patients on PPI therapy for 3 months, examining the potential additional benefits of HRW in reducing GERD symptoms and modulating systemic OS. This study adopts open-label design without strict blinding, and patients’ daily diet and eating habits were not controlled, bringing confounding interference to oxidative stress indicators. The study used various measures, including GERD-specific quality-of-life questionnaires, reactive oxygen metabolite tests, biological antioxidant potential tests, and quantification of O_2_^−^, NO, and malondialdehyde levels, to comprehensively evaluate oxidative and nitrosative stress and antioxidant capacity. The results showed that patients receiving HRW supplements experienced remarkable improvements in oxidative balance, suggesting stronger antioxidant defense and reduced oxidative damage. These findings suggest that combining PPIs with HRW may not only improve symptomatic outcomes but also restore oxidative homeostasis, potentially enhancing the overall QoL for GERD patients (Table 6).

**TABLE 6:** The effect of H_2_ intervention in other diseases clinical trials.

Disease type	Sample size	Routes of H_2_ delivery	Duration	Main effects	Authors and year
GERD	84	Drinking HRW	3 months	Heartburn↓, Regurgitation↓, NO↓, Quality of life↑, oxidative stress↓	Franceschelli et al. (2018)
HD	262	Hydrogen-rich dialysis fluid	12 months	Fatigue↓, Pruritus↓	Nakayama et al. (2017)
HD	309	Hydrogen-rich dialysis fluid	3.28 years	Antihypertensive agents↓ The prognosis of chronic HD patients↑	Ostojic et al. (2014)
HD	8	Hydrogen-rich dialysis fluid	1 day	Reversibly oxidized albumin↓	Terawaki et al. (2014)
Dry eye	54	Drinking H_2_ producing milk	3 weeks	fTBUT↑, 8-OHdG concentration in tears↓	Kawashima et al. (2019)
Phaco	32	H_2_ perfusion fluid irrigation	Perioperative	Corneal endothelial damage↓	Igarashi et al. (2019)
CHAD	50	Drinking HRW	60 days	Inflammatory response↓, Oxidative stress↓	Zhang et al. (2025)
Healthy volunteers	34	Drinking HRW	1 day	FMD↑	Sakai et al. (2014)
Aging	40	Drinking HRW	6 months	Brain choline↑, NAA levels ↑, Brain creatine at the right parietal white matter ↑, chair stand performance ↑	Zanini et al. (2021)
PMS	65	Drinking HRW	3 months	WHOQOL-BREF↑, PMSS↓	Aker et al. (2024)

### 11.2 H_2_ and hemodialysis

Chronic inflammation is a major complication in patients on hemodialysis (HD), and current therapeutic strategies to manage this condition are insufficient (Zhang et al., 2025c; Hou and Chen, 2025). Nakayama et al. (2017) developed an H_2_-rich dialysis fluid by electrochemically infusing H_2_ into reverse osmosis water used for the dialysis concentrate. In a self-controlled before-and-after study (Level 6 evidence), 21 stable HD patients switched from conventional dialysis to H_2_-enriched dialysis fluid three times a week for 6 months. All participants received sequential intervention without parallel control group, and seasonal physiological changes may affect blood pressure and inflammatory indicators. The treatment was well tolerated, with no adverse clinical events reported. Systolic blood pressure (SBP) was noticeably lower before and after dialysis, and inflammation markers, such as plasma MCP-1 and myeloperoxidase, were notably decreased. A later long-term study (Level 3 evidence) by Nakayama et al. (2018) in 2018 examined 161 patients treated with H_2_-enriched dialysis fluid compared to 148 patients on standard dialysis over 3.3 years. The two groups had inconsistent basic cardiovascular medication regimens, which interfered with hypertension incidence statistical results. The research showed a lower rate of hypertension in the H_2_-treated group, along with low antihypertensive medication requirements. These findings indicate that H_2_-enriched dialysis fluid can attenuate systemic inflammation and improve blood pressure management in HD patients. A clinical trial involving eight HD patients examined the effects of H_2_ intervention on OS by measuring blood levels of glutathione (GSH), H_2_O_2_, and albumin redox status. This is an ultra-small-sample single-arm observational study (Level 6 evidence), lacking statistical reliability for oxidative stress outcome verification. After treatment with H_2_-enriched dialysis fluid, patients showed decreased GSH levels and lower OS markers, indicating improved systemic antioxidant capacity (Terawaki et al., 2014). These studies suggest that adding H_2_ to dialysate can reduce chronic inflammation and OS in HD patients. Using H_2_-enriched solutions presents a potential supplementary method to enhance clinical outcomes and could be a tentative approach for managing uremia and related complications.

### 11.3 H_2_ and ophthalmic diseases

The eye is one of the most vital sensory organs, and many ophthalmic disorders, including cataracts and dry eye disease, are closely associated with elevated OS and inflammatory activity. Although suppressing oxidative processes and lowering excessive ROS may provide protective or therapeutic benefits, the efficacy of conventional antioxidant treatments remains limited (Chaudhary et al., 2025; Wei et al., 2024). H_2_, with its high diffusibility and selective neutralization of cytotoxic ROS, possesses several biological advantages that support its potential as an ocular therapeutic agent. Kawashima et al. (2019) conducted a randomized, double-blind clinical study (Level 2 evidence) demonstrating that consumption of milk formulated to enhance intestinal H_2_ production for 3 weeks raised exhaled H_2_ levels in individuals with dry eye disease compared to controls.This high-quality trial only adopts oral indirect H_2_ intervention, failing to compare curative effect with direct local ocular H_2_ administration. This intervention also improved visual display terminal–associated dry eye symptoms. Participants in the H_2_-producing group experienced longer tear film breakup times, and women under 45 years of age showed reduced concentrations of 8-hydroxy-2′-deoxyguanosine in tear fluid. Exhaled H_2_ levels were negatively correlated with OS markers and positively correlated with tear film stability. These findings imply that H_2_ may alleviate dry eye symptoms by reducing excessive OS, thus supporting tear film homeostasis. These data introduce a novel and practical method for integrating H_2_-based therapies into clinical dry eye treatment. In another study, a prospective, randomized, double-blind clinical trial (Level 2 evidence) evaluated the protective role of H_2_-rich intraocular perfusion fluid in 32 patients undergoing bilateral phacoemulsification cataract surgery. Paired-eye design eliminates individual differences, but short postoperative observation cycle cannot verify long-term corneal endothelial recovery status.

Each patient received H_2_-enriched perfusion fluid in one eye and standard perfusion fluid in the other eye. The eye treated with H_2_-rich perfusate showed notably smaller reductions in corneal endothelial cell density at postoperative days 1, 7, and 21, while postoperative visual acuity, intraocular pressure, and anterior chamber depth remained similar between the two eyes. These findings suggest that H_2_ can attenuate endothelial injury induced by phacoemulsification and does not negatively impact postoperative recovery (Igarashi et al., 2019). Therefore, the use of H_2_-enriched intraocular perfusion solutions appears prospective for patients who are at increased risk of corneal endothelial compromise during cataract surgery. However, further standardization regarding formulation, storage stability, and clinical application protocols is needed before routine adoption.

### 11.4 H_2_ and altitude sickness

Chronic high-altitude disease (CHAD) is defined by secondary polycythemia, hypoxic pulmonary hypertension, and increased blood viscosity. Previous research shows that targeting OS and inflammation is crucial for managing CHAD (Swenson, 2022; Garrido et al., 2021; Fan et al., 2025). Due to its known antioxidant and anti-inflammatory properties, HRW has been explored as a potential treatment option. From May to August 2024, an 8-week, randomized, double-blind, placebo-controlled, parallel-group trial (Level 2 evidence) was conducted with 50 CHAD patients. This trial implemented unified high-altitude living environment, but individual hypoxia adaptation ability varies greatly, weakening intervention efficacy difference; the 60-day intervention cycle may be insufficient to induce marked biomarker changes. Participants were assigned to either the HRW group or the placebo water group for a 60-day intervention. Researchers measured OS biomarkers, inflammation-related immune cell profiles, and whole-transcriptome changes before and after treatment to elucidate the molecular effects of HRW in CHAD. Although the HRW group showed a downward trend in OS and inflammation markers after 60 days, these changes were not statistically marked (Zhang et al., 2025d). These findings suggest that HRW may confer protective benefits in CHAD and could serve as a supplementary therapeutic approach.

### 11.5 H_2_ and endothelial dysfunction and cardiovascular disease

Endothelial dysfunction is a key early event in cardiovascular disease (CVD), and ROS play a central role (Li et al., 2025), ROS impair vasomotor function by neutralizing endothelium-derived relaxing factor nitric oxide (•NO) to form the more harmful peroxynitrite (Yan R. et al., 2024). The main sources of ROS in blood vessels are NADPH oxidase (NOX) family proteins, including NOX1, NOX2, NOX4 and NOX5, which produce superoxide and H2O2 (Singh et al., 2024). Shear stress caused by changes in blood pressure and flow activates NOX1, NOX2 and NOX3, leading to superoxide generation; this superoxide rapidly removes excess •NO inside cells to produce peroxynitrite, thereby inhibiting •NO-mediated vasodilation (Singh et al., 2024). Peroxynitrite also suppresses endothelial nitric oxide synthase (eNOS) activity, reducing NO production, and inactivates the eNOS cofactor tetrahydrobiopterin (BH4), causing eNOS uncoupling that further produces superoxide (Singh et al., 2024). The resulting redox imbalance between •NO and superoxide eventually leads to endothelial dysfunction (Yan R. et al., 2024). Accumulated oxidative stress and inflammation can cause endothelial dysfunction and promote atherosclerosis and CVD (Yan R. et al., 2024). Endothelium-dependent relaxing factors such as •NO, EDHF and prostacyclin also play important roles in dietinduced vascular dysfunction (Singh et al., 2024). Moreover, H_2_O_2_ has dual effects on vascular function, while the ·OH derived from its breakdown impairs endothelial function (Singh et al., 2024). Rheumatoid arthritis (RA) is associated with an increased risk of CVD, mainly because RA and coronary artery disease (CAD) share inflammatory pathways, with free radicals and pro-inflammatory cytokines being key links (Singh et al., 2024; Li et al., 2025). These inflammatory mechanisms affect both the vascular endothelium and joint tissues (Singh et al., 2024). Oxidation of LDL-C is an important step in atherosclerotic plaque formation and raises CVD risk (Singh et al., 2024; Li et al., 2025). Oxidative stress upregulates adhesion molecules such as VCAM-1, ICAM-1 and E-selectin, and activates pro-inflammatory cytokines including TNF-α, IL-1 and IFN-γ, all of which promote atherosclerosis (Singh et al., 2024; Li et al., 2025). Upregulation of TNF-α alone can cause vascular dysfunction and atherosclerosis, and anti-TNF-α therapy slows atherosclerosis progression in RA patients, suggesting that shared TNF-α/ROS inflammatory pathways are involved (Singh et al., 2024). H_2_ can improve endothelial function and cardiovascular outcomes through several routes. One clinical study showed thatH_2_ therapy considerably increased flow-mediated dilatation (FMD) in healthy volunteers: in the high-concentration H_2_ group, FMD rose from 6.80% ± 1.96%–7.64% ± 1.68%, whereas in the placebo group it fell from 8.07% ± 2.41%–6.87% ± 2.94% (Sakai et al., 2014) (Level 2 evidence). This suggests that H_2_ protects vascular tissues from damage induced by shear stress and hydroxyl radicals while preserving the beneficial effects of •NO on vasomotor function (Sakai et al., 2014). In a rat model of myocardial infarction, inhaling 2% H_2_ for 3 h daily over 28 days considerably improved cardiac function and reduced the area of fibrosis; *in vitro* experiments confirmed that H_2_ alleviated hypoxia-induced damage to cardiac cells and inhibited angiotensin II-induced migration and activation of cardiac fibroblasts (Nie et al., 2021). Work by Slezak’s group further demonstrated the protective effects of H_2_ in hypoxic post-conditioning, radiation-induced heart injury and acute cardiac injury (Singh et al., 2024). Given that oxidative stress can worsen systemic inflammation and thereby impair cardiomyocytes, β-cells, neurons and endothelium, the potential protective actions of H_2_ deserve further investigation (Singh et al., 2024).

### 11.6 H_2_ and aging

Age is a key factor in the development of many major non-communicable diseases, i.e., cardiovascular and metabolic disorders, neurodegenerative conditions, and cancers (Campisi et al., 2019; Li et al., 2024). In 2025, Kroemer et al. (2025) proposed fourteen hallmarks of aging: genomic instability, telomere attrition, epigenetic alterations, loss of proteostasis, dysregulated nutrient sensing, mitochondrial dysfunction, cellular senescence, stem cell exhaustion, altered intercellular communication, impaired autophagy, chronic inflammation, gut microbiota dysbiosis, extracellular matrix alterations, and psychosocial isolation. Owing to its low molecular weight, H_2_ is rapidly absorbed by tissues, readily crosses cell membranes, and diffuses quickly through the cytoplasm, nucleus, and blood–brain barrier, thereby showing marked efficacy in preventing and treating various aging-related diseases. Through its antioxidant effects, H_2_ helps maintain genomic stability (Kato et al., 2012), slow cellular senescence (Sakai et al., 2019), and participate in histone modification (Sobue et al., 2017) and telomere maintenance (Zanini et al., 2021). Moreover, H_2_ prevents inflammation, counteracts the imbalance between pro- and anti-inflammatory cytokines, and alleviates inflammaging. It also modulates the mammalian target of rapamycin (mTOR) system (Zhuang et al., 2020), autophagy (Meng et al., 2025), apoptosis (Khiji et al., 2024; Dhillon et al., 2024), and mitochondrial function (Gvozdjáková et al., 2020). These effects of H_2_ on aging hallmarks contribute to decelerating the aging process. HRW has been reported to exert anti-inflammatory effects, modulate metabolic processes, and reduce wrinkle formation, suggesting potential benefits for age-related physiological changes (Fu et al., 2022; Atiakshin et al., 2023). A randomized, controlled pilot study (Level 2 evidence) (Zanini et al., 2021) examined the effects of a 6-month HRW intervention on molecular and physical markers of aging in adults aged 70 years or older. This pilot study has limited sample size, and elderly combined basic diseases interfere with telomere and epigenetic index detection results. Forty participants were randomly assigned to drink either 0.5 L of HRW or control water daily during the study. The results indicated a positive effect of HRW on telomere dynamics, with telomere length increasing in the HRW group but decreasing in the control group. At the 6-month check, both groups showed increased expression of DNA methylation markers; however, the increase was significantly greater in the HRW group. A clear treatment-time interaction trend was also observed, characterized by rising methylation levels in the HRW group and declining levels in the control group. HRW consumption also improved several neurochemical markers, including higher levels of brain choline and N-acetylaspartate (NAA) in the left frontal gray matter, increased creatine in the right parietal white matter, and higher NAA in the medial gray matter of the right parietal lobe. Moreover, chair-stand performance improved considerably compared to the control group. Due to its wide range of biological effects, H_2_ represents a tentative candidate worthy of further exploration for potentially alleviating aging-related functional decline and telomere attrition. However, aging is a complex, multifactorial process, and the precise mechanisms by which H_2_ exerts its effects on human aging remain to be further investigated.

### 11.7 H_2_ and premenstrual syndrome

Premenstrual Syndrome (PMS) is a common emotional disorder among women. Inflammation and oxidative stress are regarded as potential causes of PMS (Cheng M. et al., 2025). HRW has the effects of alleviating inflammatory responses and antioxidation (Köktürk et al., 2022). In a randomized controlled clinical study (Level 2 evidence), researchers randomly divided 65 subjects into a HRW group (33 subjects) and a control group (32 subjects) to investigate the effect of drinking HRW on premenstrual syndrome. The trial adopts open-label design without blinding, and individual menstrual cycle regularity and sleep status act as uncontrolled confounding factors. The HRW group was required to drink 1.5–2 L of HRW daily, while the control group drank the same volume of ordinary water. The intervention started from day 16 of the menstrual cycle and continued until day 2 of the next menstrual cycle for three consecutive menstrual cycles. The results showed that the average PMSS scores of the intervention group were significantly lower than those of the control group at the first and second follow-ups (P < 0.05). At the first follow-up, the intervention group had significantly higher scores in the physical health and psychological health domains of the WHOQOL-BREF than the control group (P < 0.05), indicating that HRW intake can alleviate the severity of premenstrual symptoms and improve the quality of life in physical and psychological domains (Aker et al., 2024). The advantages and physiological effects of H_2_ provide a new strategy for the management of premenstrual syndrome.

H_2_ exerts universal selective antioxidation, anti-inflammation and immune-regulating effects on multiple extrapulmonary and extrapancreatic organs, covering digestive, renal, ocular, altitude-adaptive, senescent and reproductive endocrine systems. Current evidence includes Level 2 high-quality double-blind RCTs, Level 3 controlled trials and Level 6 small-sample observational studies. General limitations of included studies involve non-unified H_2_ administration dosage and cycle, single population inclusion, lack of multi-index long-term monitoring, and undisturbed living condition control. Further stratified clinical trials targeting different disease severity and population characteristics are required to standardize peripheral H_2_ intervention regimens and expand its multidisciplinary clinical application scope.

## 12 Evidence hierarchy of hydrogen therapy

According to study design and evidence quality, relevant publications on H_2_ therapy for diverse diseases were classified into seven tiers (Figure 1). To date, no official clinical consensus has been established, and no case-control studies (Level 4) were retrieved in the present literature. Systematic reviews or meta-analyses (Level 1) are currently absent; therefore, randomized controlled trials (Level 2) represent the highest level of clinical evidence identified, covering tumors, metabolic disorders, neurological diseases, dermatoses, respiratory diseases, arthropathy and other systemic conditions. The strength and maturity of available evidence vary markedly across disease types: metabolic syndrome, non-alcoholic fatty liver disease and diabetes are well supported by Level 2 randomized controlled trials with adequate clinical data, whereas studies on pemphigus vulgaris, acute erythematous skin diseases and skin anti-wrinkle therapy rely merely on Level 6 observational data (case series/case reports), remaining preliminary and lacking sufficient reliability for clinical application. Prospective cohort studies (Level 3) primarily focus on tumors, skin diseases, respiratory disorders and patients undergoing hemodialysis, while single-arm observational studies (Level 6) are also common in these fields. The antioxidant, anti-inflammatory and metabolic regulatory effects of H_2_ have only been validated via Level 7 cellular and animal experiments. Overall, the clinical evidence for H_2_ therapy is uneven across different diseases; current data strongly support its application in metabolic disorders, while its clinical efficacy for the aforementioned skin conditions has not been fully verified, and related mechanistic investigations are still limited to Level 7 preclinical research.

## 13 Synergistic effects of hydrogen with existing therapies

Due to the multi-target mode of action of H_2_, the therapeutic efficacy of H_2_ monotherapy may be limited. Therefore, combination therapy is required to manifest the vital value of H_2_ in the field of disease treatment. For instance, the combination of H_2_ with the PI3K inhibitor LY294002 can enhance the inhibitory effect on A549 cells (Jiang et al., 2018). In an orthotopic gastric cancer animal model, orally administered MgB_2_ nanosheets that can release H_2_ via feed intake release H_2_ *in situ* under the acidic gastric juice environment to directly act on gastric tumour cells, exerting a synergistic effect with the chemotherapeutic drug doxorubicin to enhance therapeutic efficacy. Meanwhile, the cardiac adverse reactions induced by doxorubicin are effectively alleviated, which truly achieves the effect of enhancing efficacy and reducing toxicity (Fan et al., 2019). In clinical practice, given that H_2_ can improve immune function in tumour patients, the combination of H_2_ with PD-1 antibodies has been found to improve the clinical efficacy of immunotherapy and prolong patients’ survival time (Akagi and Baba, 2020). Additionally, studies have indicated that the combination of H_2_ and therapeutic hypothermia (TH) can improve short-term neurological outcomes in asphyxiated piglets and shorten the duration of status epilepticus within 24 h after hypoxic-ischaemic injury (Tsuchiya et al., 2024). A Randomized Controlled Trial revealed that H_2_ ventilation combined with therapeutic hypothermia increased cerebral blood volume and decreased cerebral haemoglobin oxygen saturation compared with therapeutic hypothermia alone, which may reflect elevated cerebral blood flow to meet the increased oxygen demand of surviving neurons (Nakamura et al., 2023). Compared with therapeutic hypothermia management alone, H_2_-combined therapeutic hypothermia management improved neurological prognosis in patients with out-of-hospital cardiac arrest of cardiac origin; regrettably, H_2_-combined therapeutic hypothermia management did not improve survival rates (Tamura et al., 2024). Furthermore, Chang et al. (2023) found that hyperbaric oxygen combined with HRS could prevent acute lung injury. Studies have shown that hydrogen- and hyperoxia-enriched saline inhibits lipopolysaccharide-induced lung injury in C57BL/6 mice by regulating the NF-κB/NLRP3 signalling pathway (Fan et al., 2022). Co-administration of HRW with JWH-133 (a selective cannabinoid receptor type 2 agonist) or pregabalin produced a stronger analgesic effect. The combination therapy also mitigated oxidative stress, inflammation, maladaptive neural changes and nociceptive pathways activated by peripheral nerve injury (Tort et al., 2025). Zhu et al. (2025) demonstrated that H_2_ synergizes with mild hyperthermia to improve the wound microenvironment of pressure ulcers, promote the proliferation and migration of skin cells, inhibit cell apoptosis, alleviate inflammatory responses, and facilitate neovascularization, thereby supporting the healing of pressure ulcer wounds. Yoshiura et al. found that the combined application of H_2_ and Mg attenuates key pathophysiological processes associated with early brain injury and delayed cerebral ischaemia after subarachnoid haemorrhage, potentially improving prognosis by reducing depolarization events and their associated ischaemia. H_2_ treatment may provide an early protective effect prior to the onset of Mg-mediated actions (Yoshiura et al., 2025). These findings suggest that H_2_ is a prospective adjuvant therapeutic agent that may help improve therapeutic efficacy and reduce drug-related adverse reactions.

## 14 Summary and prospect

Current preclinical and clinical studies have confirmed that H_2_ exerts therapeutic effects on a variety of systemic diseases, including tumors, metabolic disorders, neurological diseases, dermatological diseases, respiratory diseases, and inflammatory bone and joint diseases, etc. With a low molecular weight and high permeability, H_2_ can penetrate subcellular structures. Its biological functions mainly include selective antioxidant activity, anti-inflammatory effects, regulation of cell proliferation, apoptosis and differentiation, as well as modulation of mitochondrial stress and epigenetic modification, etc., thereby regulating the pathological processes of various diseases. Preliminary studies have yielded positive outcomes, whereas multiple bottlenecks still restrict the clinical translation of H_2_ therapy. Mechanistically, hydroxyl radical scavenging cannot fully explain the diverse biological functions of H_2_. Its core molecular targets, key signaling pathways, and disease-specific regulatory mechanisms remain unclear. Whether H_2_ exerts broad-spectrum therapeutic effects or acts only on specific disease subtypes needs further verification. Clinically, most studies are limited by small sample sizes, single-center designs, inadequate blinding, and inconsistent intervention parameters, with a lack of long-term follow-up data. Most available clinical evidence is derived from East Asian populations, which limits the generalizability of the results. In addition, tumor heterogeneity and individual disease differences further affect the efficacy and clinical application of H_2_ treatment. The widespread clinical application of H_2_ is also restricted by practical technical and safety limitations. High-concentration H_2_ is flammable and explosive, posing potential safety hazards. In addition, accurate quantitative administration of H_2_ faces technical difficulties, and hydrogen-rich water exhibits poor stability, making standardized and stable H_2_ intervention difficult to achieve. Moreover, H_2_ monotherapy often fails to achieve optimal therapeutic effects. The efficacy and application protocols of combined treatment with conventional drugs and physical therapies still lack systematic validation.

Numerous studies have verified the protective effects of H_2_ under various pathological conditions (Satoh, 2021; Yıldız et al., 2025; Wu et al., 2023). However, due to the lack of high-quality clinical evidence, the clinical translation of H_2_ intervention remains limited, and further clinical investigations are required to promote its clinical implementation. Several major challenges persist in this field. First, most clinical studies have small sample sizes, which reduce statistical validity and result poor generalizability. Large-scale, multicenter randomized controlled trials are needed to obtain stable and reproducible clinical conclusions. Second, the optimal dosage, concentration and intervention duration of H_2_ have not been systematically standardized. These parameters vary across different diseases, while relevant comparative studies are insufficient. Third, data regarding the long-term safety and efficacy of H_2_ therapy are inadequate. Chronic diseases usually require prolonged treatment cycles, emphasizing the necessity of long-term follow-up evaluation. Most studies included in this review, especially those with small sample sizes and short treatment courses, only report observational changes in immune indicators and biomarkers. Alterations in cytokines, oxidative stress markers and immune cell subsets cannot directly confirm definitive clinical outcomes such as tumor regression, prolonged survival or reduced recurrence rates. Current findings are mostly correlative rather than causal. Future prospective studies adopting standardized quantitative biomarkers and long-term clinical endpoints are essential to clarify the causal relationship between H_2_ intervention and clinical efficacy.

Future basic research should further explore the molecular targets and disease-specific signaling mechanisms of H_2_ and clarify its biological functions beyond antioxidant regulation. It is necessary to develop efficient and stable H_2_ delivery and storage systems to resolve hydrogen dissipation, inaccurate administration and potential safety risks. Exploring the synergistic effects of H_2_ combined with conventional therapies can improve treatment efficacy and expand the scope of clinical application. In conclusion, H_2_ displays tentative clinical therapeutic potential and could possibly deliver mild-to-moderate clinical benefits for certain patient groups. Rigorously designed, high-quality clinical trials together with in-depth mechanistic research are critical to accelerate the clinical translation and standardized application of H_2_ therapy.
